# Diversity time-period and diversity-time-area relationships exemplified by the human microbiome

**DOI:** 10.1038/s41598-018-24881-3

**Published:** 2018-05-08

**Authors:** Zhanshan (Sam) Ma

**Affiliations:** 1 Computational Biology and Medical Ecology Lab, State Key Laboratory of Genetic Resources and Evolution, Kunming Institute of Zoology, Chinese Academy of Sciences, Kunming, 650223 China; 2 Center for Excellence in Animal Evolution and Genetics, Chinese Academy of Sciences, Kunming, 650223 China

## Abstract

We extend the ecological laws of species-time relationship (STR) and species-time-area relationship (STAR) to general diversity time-period relationship (DTR) and diversity-time-area relationship (DTAR), and test the extensions with the human vaginal microbiome datasets by building 1460 DTR/DTAR models. Our extensions were inspired by the observation that Hill numbers, well regarded as the most appropriate measure of alpha-diversity and also particularly suitable for multiplicative beta-diversity partitioning, are actually in the units of *effective* species, and therefore, should be able to substitute for species in the STR and STAR. We found that the traditional power law (PL) model is only applicable for DTR at diversity order zero (*i.e*., species richness); at higher diversity orders (*q* = 1–4), the power law with exponent cutoff (PLEC) and power law with inverse exponent cutoff (PLIEC) are more appropriate. In particular, PLEC has an advantage over PLIEC in predicting maximal accumulation diversity (MAD) over time. In fact, with the DTR extensions, we can construct DTR and MAD profiles. To the best of our knowledge, this is the first comprehensive investigation of the DTR/DTAR in human microbiome. Methodologically, our DTR/DTAR profiles can characterize general diversity scaling beyond species richness, covering both alpha- and beta-diversity regimes across different diversity orders.

## Introduction

Although not as well known as species-area relationship (SAR), species-time relationship (STR) first conjectured by Preston^1^, is an equally important ecological law. We believe that the less conspicuous position of the STR in ecology is largely due to the relative scarcity of longitudinal (temporal) datasets of ecological communities, rather than being theoretically less interesting or practically less useful. Indeed, as demonstrated below, STR can be more complex than its spatial counterpart because the accumulation of species richness over time can be fluctuating, not necessarily monotonically increasing. According to Preston’s^1^ ergodic conjecture, space and time (especially evolutionary time) should be equivalent in terms of the accumulation of species, and we will try to shed light on this classic hypothesis. Rosenzweig^2^ tested Preston’s STR conjecture with several large-scale survey datasets, and further developed a joint version of time and area, *i.e*., STAR (species-time-area relationship) model. In recent years, there have been renewed interests in STR and STAR, especially the discussion on their mechanisms [e.g., Alder *et al*., Ulrich, and McGlinn & Palmer]^3–7^.

Extensive theoretical and empirical studies on SAR and STR have been performed in macro-ecology (*e.g*., Preston^1^, Rosenzweig^2,8^, Lomolino^9^, Tjørve, Drakare *et al*., Harte *et al*., He & Hubbell, Sizling *et al*., Storch *et al*., Triantis *et al*., Helmus *et al*., Alder *et al*., Ulrich, McGlinn & Palmer)^1–7,9–20^. Nevertheless, the study of microbial SARs/STRs^21–32^ is another story. For example, the first investigation of a microbial SAR was not reported until this century (Green *et al*. & Horner-Devin *et al*., Bell *et al*.)^33–35^, and the first study of a microbial STR is even later (Oliver *et al*., Shade *et al*.)^21,22^. This is because either microbial SAR or STR research is heavily dependent on the DNA sequencing technology, which was not widely available until recent years. In the last decade, further advances in the next generation sequencing (NGS) technology, more recently the third generation sequencing technology (3GS), and most directly, the sequencing-supported metagenomics technology have revolutionized the studies of microbial community ecology. Several studies of the microbial SAR and STR have been performed (*e.g*., Noguez *et al*., Peay *et al*., van der Gast *et al*., Costello *et al*., Lozupone *et al*., Lyons *et al*., Jones *et al*., Oliver *et al*., Ristova *et al*., Zinger *et al*.)^21,23–32^, but compared with their counterparts in macro-ecology the number of studies on microbial STR is nearly negligible.

Even with the rapidly expanding revolutionary advances in microbial community ecology, to the best of our knowledge, testing of STR with the human microbiome data has not been systematically investigated except for perspective general discussions (Ma *et al*., Oliver *et al*., Shade *et al*.)^21,22,36^. In this article, we report our comprehensive test and extensions of the traditional STR with recently available dataset from a longitudinal study of the human vaginal microbiome (Gajer *et al*.)^37^.

Methodologically, we extend the traditional STR and STAR models to the general diversity-time relationship (DTR) and diversity-time-area relationship (DTAR) models, respectively, by using the Hill Numbers as a general diversity metric system, including both *alpha-* and *beta*-diversity. The choice of Hill numbers not only makes our study more comprehensive than existing studies of SAR/STR, but also makes it possible to extend the traditional STR to both alpha- and beta-diversities beyond species richness (*i.e*., the zero-order diversity in terms of the Hill numbers). We take advantage of the recent consensuses that Hill numbers offer the best measure of alpha- diversity and that multiplicative beta-diversity partitioning of the Hill numbers is more appropriate than the alternative additive partition (Ellison, Chao *et al*., Jost, Chiu *et al*., Chao & Jost)^38–44^. As a side note, as explained in following section of methods, the time (*T*) we discuss in this article should be termed *time period* or *period*, because what is usually accumulated is a *period* of time, rather than time points. That is, more appropriate terms should be *diversity time period* or *diversity period*. In consider of the traditional usage of *species time* in ecology, we use the term *diversity time* and *diversity-time period* interchangeably. Accordingly, the acronym DTR may be interpreted as either diversity time relationship or diversity time-period relationship in this article.

To the best of our knowledge, this is the first extension of the STR/STAR to general diversity-time scaling (DTR/DTAR) beyond species-richness level in terms of the Hill numbers. Previously, a few authors, notably Helmus & Ives^45^, Mazel *et al*.^46^ have successfully extended the SAR to phylogenetic and functional diversities. Their extensions not only verified the applicability of SAR models beyond traditional species richness, but also found important applications in identifying more comprehensive conservation hotspots and predicting the impacts of habitat loss. However, we are not aware of any similar extensions to STR in the existing literature. We expect that our methodological extensions of STRs/STARs to general Hill numbers based DTRs/DTARs should not only enrich the theoretical modeling of the diversity scaling in terms of more comprehensive diversity profiles, but also offer important novel insights for their ecological applications. In addition, the Hill numbers, as the metric for a diversity profile, have advantages lacked by other existing diversity measures. For instances, the Hill numbers effectively capture the essential properties (such as rarity *vs*. commonness) of species abundance distribution (SAD) by computing the entropy at different orders (non-linearity levels) (Ellison, Chao *et al*., Jost, Chiu *et al*., Chao & Jost)^38–44^. These advantages are naturally carried over to our general DTR/DTAR extensions.

Theoretically, the proposed DTR/DTAR can characterize the temporal dynamics/spatial-temporal dynamics of community diversity. Furthermore, with our derived formula, the MAD (maximal accumulation diversity) over time can be predicted. Practically, Oliver *et al*.^21^ emphasized that future importance of studying the microbial STR includes to distinguish between anthropogenic perturbations and underlying natural dynamics, and to offer ecological insights for clinical benefit in fighting bacterial infections. Their point makes particular sense in the case of the human vaginal microbiome (HVM) because the DTR can potentially be utilized to assess the bacterial temporal turnover or diversity dynamics of the HVM.

## The Methods—Extensions from STR/STAR to DTR/DTAR

We use the following definitions and computational procedures to design, conduct, and interpret DTR and DTAR modeling analyses.

### Definitions of the alpha- and beta-diversity measured in the Hill numbers

We adopt the Hill numbers to measure both alpha- and beta-diversities, and adopt *multiplicative partition* of the Hill numbers as beta diversities.

The Hill numbers, originally introduced as an *evenness* index in economics by Hill^47^ who was apparently inspired by Renyi’s^48^ general entropy, did not receive the attention it deserves in ecology until recent years (*e.g*., Jost, Chao *et al*.)^39–42^:

1 $${}^{q}D={(\sum _{i=1}^{S}{p}_{i}^{q})}^{1/(1-q)}$$

where *S* is the number of species, *q* is the order number of the Hill numbers.

The Hill number is undefined for *q* = 1, but its limit as *q* approaches 1 can be derived as:

2 $${}^{1}D=\mathop{\mathrm{lim}}\limits_{q\to 1}{}^{q}D=\exp (-\sum _{i=1}^{s}{p}_{i}\,\mathrm{log}({p}_{1})).$$

The parameter *q* determines the sensitivity of the Hill number to the relative frequencies of species abundances. When *q* = 0, the species abundances do not count at all and ^0^*D* = *S, i.e*., species richness. When *q* = 1, ^1^*D* equal the *exponential* of Shannon entropy, and is interpreted as the number of typical or common species in the community. When *q* = 2, ^2^*D* equal the reciprocal of Simpson index, *i.e*.,

3 $${}^{2}D=(1/\sum _{i=1}^{S}{p}_{i}^{2}),$$

which is interpreted as the number of dominant or very abundant species in the community (Chao *et al*.)^39^.

The general interpretation of diversity of order *q* is that the community contains ^*q*^*D* = *x equally* abundant species, or the so-termed *effective* number of species (or species equivalents such as OTUs). When the species are equally abundant, the relative abundance does not weigh in the computation of the Hill numbers, and consequently, the Hill numbers are determined by the number of *effective* species completely. Note that, although the assumption of equal relative abundance is rather rarely satisfied and the computation of Hill numbers is usually dependent on both the number of species and their abundances, the principle underlying the equivalence between the *effective number of species* and the Hill numbers still holds regardless of the assumption. It is this equivalence principle that inspired our extensions from STR to DTR (this article) and from SAR to DAR (Ma)^49^.

The diversity of a community can be measured with a series of Hill numbers, possibly plotted on a single graph as a ‘continuous’ function of the parameter *q*. Chao *et al*.^39^ termed the series of plots “*community diversity profile*” that characterizes the species abundance distribution (SAD) of a community and offers complete information on its diversity. As explained later, we extend the concept of diversity profile to the DTR profile and MAD (maximal accumulation diversity) profile.

Recent advances (*e.g*., Jost, Ellison, Chao, Chao *et al*., Chiu *et al*., Chao & Jost)^38–44^ have made a convincing case that Hill numbers and multiplicative beta-partition offer to date the most generally consistent and appropriate, yet simple solution to measure biodiversity. Jost^44^ demonstrated that the partition of Hill numbers into independent alpha-diversity (within community) and beta-diversity (between communities) is necessarily multiplicative, *i.e*.,

4 $${}^{q}D_{\beta }={}^{q}D_{\gamma }/{}^{q}D_{\alpha }$$

This beta-diversity derived from the above partition takes the value of 1 if all communities are identical, and the value of *N* (the number of communities) when all the communities are completely different from each other (no shared species). In general, the beta-diversity defined in this multiplicative partition is, with Jost^44^ words, “*the effective number of completely distinct communities*.”

In this article, we compute diversities until *q* = 4, *i.e*., to the fourth order, which includes traditional *species richness* (*q* = 0), the exponential of Shannon index (*q* = 1), the reciprocal of Simpson index (*q* = 2) and two additional sets of diversity indexes for *q* = 3 and 4, respectively.

### The DTR (Diversity Time Relationship) models

We were inspired by the observation that Hill numbers, well regarded as the most appropriate measure of the alpha-diversity, are actually in the units of species; indeed, they are referred to as the *effective number of species* or as *species equivalents* (such as OTUs), as explained in the previous section. Furthermore, Hill numbers capture the essential distribution properties of species abundances (rarity *vs*. commonness) by computing the entropy at different orders (non-linearity levels); they are particularly suitable for multiplicative beta-diversity partitioning, which is superior to its alternative—additive partitioning. Based on these inspirations, we postulate that the models of traditional STR should be extendable to model general DTR.

In consideration of the debates on the functional forms of SAR/STR, we selected three models, *i.e*., power law (PL) model as well as its two extensions, the PL with exponential cutoff (PLEC) and the PL with inverse exponential cutoff (PLIEC), from more than 20 SAR/STR models existing in the literature (Tjørve, Triantis *et al*., Williams *et al*., Whittaker & Triantis)^11–13,19,50,51^ to build the DTR models.

We assume that the basic power law (PL) function widely adopted in the STR scaling study, known as *power-law* species scaling law can be extended to model the general DTR:

5 $${}^{q}D=c{T}^{w}$$

where ^*q*^*D* is diversity in the form of *q*-order Hill numbers, *T* is *time*, and *c* & *w* are parameters. Strictly speaking, *T* should be *time period* or *period*, because what is usually accumulated is a *period* of time, rather than time points. In consider of the traditional usage of *species time* in ecology, we use the term *diversity time*. Nevertheless, obviously more appropriate terms should be *diversity time period* or *diversity period*.

We also extend two additional models, which were originally introduced to model SAR by Plotkin *et al*.^52^ and Ulrich & Buszko^53^, to investigate the DTR. The first is the *power law with exponential cutoff* (PLEC),

6 $${}^{q}D=c{T}^{w}\exp (dT),$$

where *d* is a third parameter, and exp(*dT*) is the exponential decay term that eventually overwhelms the power law behavior at very large value of *T*. The justification for adding the exponential decay term is because diversity accumulation may saturate and even decline over time, and therefore, there should be a taper-off item to reflect the reality.

Another function similar to (6) but with *Sigmoid* shape, rather than convex as (6) is the following power-law function with an *inverse exponential cutoff* (PLIEC):

7 $${}^{q}D=c{A}^{w}\exp \,(d/A).$$

PLEC has an advantage over the other two models in predicting the maximal accumulation diversity (MAD), derived below. The two models we here refer to as the PLEC and PLIEC were usually referred to as the *first* and *second persistence* model, or *persistence function*-I and *persistence function*-II, respectively (*e.g*., Tjørve).

We use the following log-linear transformed Eqns (8), (9), (10) to estimate the model parameters of Eqns (5), (6), (7), respectively:

8 $$\mathrm{ln}({}^{q}D)=\,\mathrm{ln}(c)+w\,\mathrm{ln}\,(T)$$

9 $$\mathrm{ln}({}^{q}D)=\,\mathrm{ln}(c)+w\,\mathrm{ln}\,(T)+dT$$

10 $$\mathrm{ln}({}^{q}D)=\,\mathrm{ln}\,(c)+w\,\mathrm{ln}(T)+d/T$$

where all the symbols represent the exactly same variables (parameters) as those in Eqns (5), (6), (7). These linear models are computationally simple to fit, and we use both linear correlation coefficient (*R*) and *p*-value to judge the goodness of the model fitting, either of which should be sufficient to judge the suitability of the models to data.

The taper-off parameter (*d*) in both the PLEC and PLIEC not only addresses a critique to the traditional power law for overestimating diversity (He & Hubbell)^16^, but also preserves the biological interpretations of the scaling parameter (slope *z* of SAR or *w* of STR) since *d* is primarily a revision to the other biologically less important parameter *c* (Tjørve)^13^. Furthermore, PLEC allows for the estimation of the *maximal accumulation diversity* (MAD) of the human microbiome over time times, as detailed below.

### The DTAR (Diversity Time Area Relationship) models

Rosenzweig^8^ extended the traditional STR model (5) to general STAR (species time area relationship) model, and more mechanistic discussions on STAR can be found in Alder *et al*., Ulrich and McGlinn & Palmer^3–7^. Here we further extend it to general diversity time area relationship (DTAR). Both the extensions adopt the following *bivariate power law* (BPL) model:

11 $${}^{q}D=c{A}^{z}{T}^{w}.$$

Its log-transformed linear form is:

12 $$\mathrm{ln}({}^{q}D)=\,\mathrm{ln}(c)+z\,\mathrm{ln}(A)+w\,\mathrm{ln}(T)$$

where ^*q*^*D* is the diversity measured with the Hill numbers of *q*-order, *A* is the *area*, *T* is the time, *z* and *w* are scaling exponents corresponding to *area* and *time*, respectively, *c* is a parameter that is largely determined by sampling design but with limited biological meanings.

Different from most studies in macro-ecology, where there is often a natural spatial sequence (or arrangement) among the communities sampled, the community samples in our HVM data are somewhat ‘random’ since there is not a natural order among the 32 individual subjects. To avoid the potential bias from an arbitrary order of the community samples, we totally permutate the orders of all 32 subjects under investigation, and then randomly select (without replacement) 50 orders of the communities generated from the permutation operation. That is, rather than building only one DTAR model for the dataset, we build 50 such models and take the average of their parameters as the final DTAR parameters. In the time dimension of each individual subject, we simply follow the natural (calendar) sequence of the time-series data.

Therefore, to build a DTAR model, one of the 50 randomly sampled sequences is used to determine the accumulation order of the area (individuals), and the diversity profile is then computed for each of the time-series point of each individual subject. If *D*_*ij*_ (*i* = 1, …, 32, *j* = 1, …*T*_*i*_; *T*_*i*_ is the number of times that the *i*-th subject were sampled over time series) represents the diversity of the *i*-th individual at time *j*, its area (*A*) and time (*T*) for fitting the DTAR models [Eqns (11) and (12)] would be *A* = *i*, and *T* = *j*. In this study, to avoid potential bias caused by different number of samples for different individuals (*T*_i_), we fix *T* to 25 times. Consequently, the total numbers of samples utilized to construct the DTAR models are fixed to 800 (32*25) times. Finally, the averages of the model parameters from the 50 times of DTAR fittings are adopted as the model parameters of the DTAR for the set of community samples under investigation.

### Sampling and accumulation schemes for the DTR and DTAR models

Since we need to accumulate not only time (period) for the DTR and DTAR, but also area (individual) for the DTAR, we must devise the accumulation schemes for both area and time. We adopt the Scheiner^54,55^ Type-III sampling scheme, noncontiguous quadrats grid. Strictly speaking, quadrats (the same size) are arranged in a regular but noncontiguous grid. Since the quadrat (individual subject), *i.e*., our body (habitats for our microbiome) is highly mobile, figuring out a definite spatial coordinate relationship, not to mention a grid of quadrats, is hardly possible. Nevertheless, Type-III is obviously the most natural choice we can make.

Carey^56^ extended Scheiner’s^54^ typology to species time relationships (STR): these are *nested*, *completely nested*, and *island* approaches to construct STR models depending on their definitions of time, whether it is defined as an *interval*, a *flow* or a combination of both. In our study, the length of sampling time (period) *T* is defined as the total time (period) *T* from the start of the initial survey. The scheme is the *nested* construction, and time is defined as both an interval and a flow in terms of Carey’s classification.

After selecting the sampling schemes, we need to specify the scheme to accumulate diversity based on the diversity formulae [Eqns (1–4)] listed above. Although the accumulation of species in the traditional STR is well defined and there is little ambiguity on how the species counts are computed once the scheme for time accumulation is decided, the computation of the diversity accumulation is still largely an uncharted area, and there may be more than one scheme to accumulate diversity across space or time, especially for the accumulation of beta-diversity.

To devise what we believe to be the most appropriate and also natural methods to accumulate diversity, we propose the following three principles. The first is to use the Hill numbers, or what Jost^44^ termed the *true* diversity; the second is to follow the essence of SAR and STR, as captured by the word “accumulation” or “aggregate,” *i.e*., species (diversity) are accumulated for the accumulated times (periods); the third is that the diversity scaling model should be useful for *predicting* diversity at different accumulated times periods. We consider these three principles as axioms in traditional SAR or STR and we believe that any extension from STR to DTR should not violate them.

To accumulate alpha-diversity across times, assuming there are *N* community samples, at each accumulation step *i* = 1, 2, …, *N*-1, we simply add up all the OTU lists (rows) up to the accumulation step *i* in the standard OTU table of the communities, corresponding to the first *i* community samples, until the last sample (community *N*) is added when the accumulation is completed. For each of the aggregate (accumulated) community, we compute its alpha-diversity with Eqns (1) or (2) (for *q* = 1) with the added-up OTU lists for the accumulation step. The resulted pairs of accumulated alpha-diversities and times are regressed to fit the DTR model.

To accumulate beta-diversities across times, we start the computational procedure with two local communities (samples) by using the formulae specified by Eqns (1)–(4), from which the first beta-diversity value is computed for the two initial communities. For each newly added community (sample) at the accumulation step *i* (*i = 3, 4, … N*), we simply run the same computation procedure with a pair of communities consisting of the previously pooled *i*-1 communities as one regional community and the newly added *i*-th community as the other. With each newly added local community, we obtain a new Hill numbers of beta-diversity, until all *N* communities are accumulated for their beta-diversity. The series of beta-diversities are regressed with their respective times accumulated at each step. Obviously, this accumulation scheme calculates the *accumulated beta-diversity* of *N* communities (of individuals in the case of human microbiome), *i.e*., the difference between the pooled *N*-1 communities and the last community. It is also the maximum difference among *N* communities in terms of beta-diversity.

### Statistical distribution of the DTR/DTAR parameters

We further investigate the statistical distributions of the DTR/DTAR scaling parameters [*w*, ln(*c*), *d*, *T*_max_, *D*_max_] by fitting two contrastingly different statistical distributions: the *normal distribution* and *power-law distribution*. The former depicts a largely symmetric distribution of the scaling parameters, and the latter depicts an asymmetric (long-tail) probability distribution that has some unique properties not possessed by the normal distribution. For example, the power-law distribution usually suggests heavy heterogeneity or skewed data points. It has the so-termed “no-average” property, which means that the average of the power law distribution cannot represent majority of the data points because of the highly skewed long-tail. Such information should be particular valuable for further characterizing the individualities of the human microbiome, as well as for personalized medicine (Ma *et al*.)^57,58^.

Since the information on normal distribution can be readily found in standard statistics textbook (*e.g*., Gotelli & Ellison)^59^, we only list some basic information about the power law distribution below. Power law distribution has a probability density function as follows:

13 $$p(x)=\tfrac{K-1}{{x}_{\min }}{(\tfrac{x}{{x}_{\min }})}^{-K}$$

where *x* is the random variable, *x*_*min*_ is the minimum value of *x*, *K > 1* and is the exponent or the scaling parameter of the power law distribution. *K* can be considered as a measure of asymmetricity (skewness) of the heterogeneity in the power law distribution. A comprehensive discussion on the power law distribution can be found in Clauset *et al*.^60^.

### Predicting the MAD (maximal accumulation diversity) with DTR models

PLEC [Eqn. (6)] has an advantage over the other two models in predicting the maximal accumulation diversity. Ma^49^ derived the maximum of PLEC, which can be adapted for DTR as follows:

The necessary condition for Eqn. (6) to achieve maximum is its derivative equals zero, *i.e*.,

14 $$\frac{df(T)}{dT}={({}^{q}D)}^{\text{'}}={[c{T}^{w}\exp (dT)]}^{\text{'}}=0$$

Solving Eqn. (14), when

15 $${T}_{\max }=-w/d$$

^*q*^*D* may have a maximum in the following form:

16 $$Max({}^{q}D)=c{(-\frac{w}{d})}^{w}\exp (-w)=c{T}_{\max }^{w}\exp (-w)$$

Theoretically, Eqns (15) and (16) can be utilized to predict the maximal accumulation diversity (MAD) of the human microbiome, whether it is alpha- or beta-diversity.

### Data Accessibility

The longitudinal cohort dataset of HVM used to test DTR/DATR is available at: http://stm.sciencemag.org/content/4/132/132ra52.full.

## Results

Before presenting the test results for our DTR/DTAR extensions, here we first briefly introduce the 16 s rRNA sequence datasets we used to perform the tests. The datasets we utilized are from a longitudinal study on the HVM (human vaginal microbiome) by Gajer *et al*.^37^ with 32 healthy women at reproductive age offered to date the longest and also the most comprehensive longitudinal study of microbiome dynamics, conducted with 16*s*-rRNA sequencing technology. To reduce the influence of the noises in sequence data, we filtered out the samples that have a total number of lesser than 100 16s-rRNA reads because samples with too small number of reads are often due to the failure in amplicon amplification and may introduce significant bias in binning out the OTUs from the limited number of reads.

### Diversity-Time Relationship (DTR) modeling

We construct three DTR models (PL, PLEC, and PLIEC) for each of the 32 subjects, with their time-series data of their vaginal microbial communities, respectively. A total of 960 DTR models were built. To save page space, Tables 1 and 2 only list the partial results of alpha-diversity DTR or *A*DTR (for *q* = 0, 1) and beta-diversity DTR or *B*DTR (for *q* = 0), respectively. The online *Supplementary Information* (Suppl. Tables S1A and B) report the full results for diversity order *q* = 0–4, and a detailed examination of the modeling results is also presented there. Here, we summarize some important findings as explained below.

**Table 1:** The Alpha-Diversity Time Relationship (*A*DTR) models for the individuals in the 32-healthy cohort*.

Order	Subject ID	Power law (PL)				PL with exponential cutoff (PLEC)					PL with inverse exponential cutoff (PLIEC)					*n*	PLEC Pred.	
		*w*	ln(*c*)	*R*	*p*-value	*w*	ln(*c)*	*d*	*R*	*p*-value	*w*	ln(*c*)	*d*	*R*	*p*-value		*A*_max_	*D*_max_
*q* = 0	400	0.542	2.312	0.96	0.000	0.440	2.397	0.011	0.96	0.000	0.705	1.800	0.805	0.97	0.000	29	NA	7
	401	0.565	2.669	0.93	0.000	0.608	2.632	−0.005	0.93	0.000	0.394	3.207	−0.856	0.94	0.000	30	122	140
	402	0.463	2.567	0.92	0.000	0.083	2.904	0.039	0.97	0.000	0.736	1.697	1.398	0.96	0.000	31	NA	17
	403	0.527	1.670	0.96	0.000	0.422	1.765	0.010	0.97	0.000	0.636	1.321	0.566	0.97	0.000	32	NA	5
	404	0.329	3.358	0.98	0.000	0.409	3.289	−0.008	0.98	0.000	0.266	3.558	−0.318	0.98	0.000	30	51	89
	405	0.537	2.075	0.96	0.000	0.649	1.975	−0.011	0.96	0.000	0.470	2.287	−0.341	0.96	0.000	31	59	53
	406	0.523	2.585	0.96	0.000	0.507	2.599	0.002	0.96	0.000	0.434	2.866	−0.451	0.96	0.000	31	NA	NA
	407	0.337	3.635	0.99	0.000	0.322	3.647	0.002	0.99	0.000	0.346	3.608	0.042	0.99	0.000	28	NA	76
	408	0.366	2.702	0.98	0.000	0.258	2.792	0.012	0.98	0.000	0.385	2.643	0.093	0.98	0.000	29	NA	19
	410	0.428	2.810	0.98	0.000	0.524	2.729	−0.010	0.98	0.000	0.300	3.209	−0.629	0.99	0.000	29	52	72
	411	0.366	3.282	0.91	0.000	0.623	3.066	−0.028	0.95	0.000	0.093	4.136	−1.345	0.98	0.000	29	22	80
	412	0.198	2.854	0.96	0.000	0.131	2.909	0.007	0.97	0.000	0.206	2.828	0.040	0.96	0.000	28	NA	22
	413	0.261	3.015	0.99	0.000	0.304	2.980	−0.005	1.00	0.000	0.235	3.095	−0.126	0.99	0.000	28	61	51
	414	0.349	2.861	0.96	0.000	0.495	2.729	−0.014	0.98	0.000	0.273	3.108	−0.401	0.97	0.000	32	35	55
	415	0.310	3.069	0.94	0.000	0.476	2.927	−0.017	0.96	0.000	0.157	3.554	−0.772	0.97	0.000	30	28	57
	416	0.403	2.776	0.98	0.000	0.437	2.749	−0.004	0.98	0.000	0.329	3.006	−0.358	0.99	0.000	28	109	79
	418	0.643	2.187	0.95	0.000	1.045	1.831	−0.041	0.99	0.000	0.337	3.161	−1.566	0.99	0.000	31	25	65
	420	0.656	1.946	0.97	0.000	0.380	2.169	0.031	0.99	0.000	0.865	1.297	1.011	0.99	0.000	28	NA	6
	423	0.640	2.590	0.97	0.000	0.965	2.318	−0.035	0.99	0.000	0.397	3.351	−1.198	0.99	0.000	29	28	95
	424	0.434	3.448	0.95	0.000	0.627	3.292	−0.021	0.97	0.000	0.330	3.770	−0.502	0.96	0.000	28	30	121
	429	0.602	2.401	0.97	0.000	0.885	2.163	−0.031	0.99	0.000	0.411	2.997	−0.940	0.99	0.000	29	29	70
	430	0.259	3.533	0.98	0.000	0.204	3.576	0.006	0.99	0.000	0.327	3.324	0.322	0.99	0.000	27	NA	48
	431	0.274	3.194	0.96	0.000	0.439	3.064	−0.019	0.99	0.000	0.170	3.513	−0.491	0.98	0.000	27	23	55
	432	0.472	2.783	0.94	0.000	0.127	3.072	0.037	0.99	0.000	0.715	2.022	1.198	0.98	0.000	29	NA	20
	435	0.400	2.861	0.98	0.000	0.377	2.878	0.003	0.98	0.000	0.461	2.678	0.276	0.99	0.000	25	NA	28
	436	0.212	4.450	0.98	0.000	0.291	4.379	−0.008	0.99	0.000	0.156	4.630	−0.292	0.99	0.000	32	36	170
	437	0.432	3.020	0.95	0.000	0.491	2.965	−0.006	0.95	0.000	0.473	2.885	0.221	0.95	0.000	33	82	103
	439	0.232	2.924	0.91	0.000	0.069	3.064	0.017	0.95	0.000	0.325	2.628	0.472	0.94	0.000	30	NA	21
	443	0.253	3.905	0.99	0.000	0.300	3.868	−0.005	0.99	0.000	0.222	4.000	−0.147	0.99	0.000	28	60	121
	444	0.285	3.703	0.98	0.000	0.175	3.790	0.013	0.99	0.000	0.372	3.436	0.411	0.99	0.000	27	NA	50
	445	0.274	2.765	0.98	0.000	0.298	2.745	−0.002	0.98	0.000	0.288	2.720	0.071	0.98	0.000	30	149	51
	446	0.431	2.793	0.88	0.000	0.740	2.534	−0.033	0.92	0.000	0.326	3.122	−0.519	0.89	0.000	29	22	60
	**Mean**	**0.406**	**2.898**	**0.96**	**0.000**	**0.441**	**2.869**	**0.000**	**0.97**	**0.000**	**0.379**	**2.983**	**−0.100**	**0.97**	**0.000**	**29**	**NA**	**NA**
*q* = 1	400	0.107	0.582	0.54	0.002	−0.032	0.698	0.015	0.62	0.002	0.277	0.049	0.839	0.71	0.000	29	2	2.03
	401	0.450	0.104	0.83	0.000	−0.117	0.593	0.060	0.94	0.000	0.781	−0.942	1.663	0.89	0.000	30	2	1.88
	402	0.040	0.874	0.32	0.080	−0.161	1.052	0.020	0.76	0.000	0.128	0.592	0.453	0.51	0.015	31	8	2.40
	403	0.033	0.234	0.43	0.015	−0.033	0.295	0.007	0.56	0.004	0.061	0.147	0.142	0.47	0.027	32	5	1.32
	404	−0.107	1.922	0.52	0.003	−0.397	2.172	0.030	0.79	0.000	0.010	1.552	0.590	0.60	0.002	30	13	4.68
	405	−0.024	0.362	0.15	0.408	0.203	0.161	−0.023	0.65	0.000	−0.203	0.934	−0.919	0.67	0.000	31	9	1.49
	406	0.232	0.517	0.73	0.000	−0.117	0.826	0.036	0.86	0.000	0.474	−0.253	1.237	0.84	0.000	31	3	2.24
	407	−0.326	2.532	0.85	0.000	−0.472	2.650	0.016	0.86	0.000	−0.302	2.457	0.117	0.85	0.000	28	30	4.59
	408	0.161	1.478	0.96	0.000	0.141	1.495	0.002	0.96	0.000	0.159	1.483	−0.009	0.96	0.000	29	NA	6.38
	410	0.202	0.161	0.88	0.000	0.075	0.267	0.014	0.91	0.000	0.242	0.034	0.200	0.88	0.000	29	NA	1.34
	411	0.274	1.454	0.67	0.000	0.813	1.003	−0.058	0.87	0.000	−0.231	3.036	−2.492	0.94	0.000	29	14	10.35
	412	−0.523	2.040	0.98	0.000	−0.585	2.090	0.007	0.98	0.000	−0.550	2.126	−0.133	0.98	0.000	28	84	1.09
	413	0.019	1.646	0.17	0.394	−0.204	1.827	0.025	0.83	0.000	0.144	1.257	0.605	0.60	0.004	28	8	4.97
	414	0.142	1.372	0.95	0.000	0.195	1.324	−0.005	0.96	0.000	0.105	1.491	−0.193	0.96	0.000	32	39	6.32
	415	0.070	0.727	0.33	0.074	0.395	0.447	−0.034	0.73	0.000	−0.107	1.286	−0.890	0.57	0.005	30	12	2.78
	416	0.046	0.620	0.42	0.027	−0.162	0.788	0.023	0.89	0.000	0.157	0.275	0.537	0.68	0.000	28	7	1.88
	418	0.134	0.201	0.72	0.000	0.302	0.051	−0.017	0.82	0.000	0.033	0.522	−0.516	0.78	0.000	31	18	1.86
	420	0.441	−0.255	0.77	0.000	−0.150	0.224	0.066	0.88	0.000	0.884	−1.630	2.141	0.87	0.000	28	2	1.29
	423	0.082	0.719	0.31	0.098	0.440	0.420	−0.039	0.66	0.001	−0.282	1.857	−1.792	0.82	0.000	29	11	2.85
	424	0.081	1.048	0.46	0.014	−0.230	1.300	0.035	0.87	0.000	0.325	0.290	1.181	0.87	0.000	28	7	3.00
	429	0.134	0.072	0.91	0.000	0.145	0.064	−0.001	0.91	0.000	0.169	−0.036	0.170	0.92	0.000	29	145	1.90
	430	0.224	1.941	0.88	0.000	0.286	1.892	−0.007	0.88	0.000	0.277	1.777	0.253	0.88	0.000	27	41	14.40
*q* = 1	431	0.036	1.908	0.42	0.030	0.114	1.847	−0.009	0.56	0.010	0.022	1.951	−0.065	0.43	0.089	27	13	7.56
	432	0.002	0.638	0.01	0.966	−0.450	1.017	0.049	0.77	0.000	0.165	0.129	0.802	0.35	0.174	29	9	1.60
	435	0.083	0.973	0.66	0.000	0.051	0.996	0.004	0.67	0.001	0.068	1.016	−0.065	0.67	0.002	25	NA	2.89
	436	0.144	2.444	0.85	0.000	0.157	2.432	−0.001	0.85	0.000	0.200	2.264	0.293	0.87	0.000	32	157	21.52
	437	−0.120	0.790	0.76	0.000	−0.260	0.921	0.013	0.85	0.000	0.033	0.296	0.811	0.93	0.000	33	20	1.49
	439	−0.020	1.375	0.15	0.444	−0.292	1.610	0.029	0.85	0.000	0.158	0.815	0.892	0.72	0.000	30	10	3.41
	443	0.166	1.796	0.58	0.001	0.614	1.433	−0.050	0.87	0.000	−0.097	2.612	−1.272	0.75	0.000	28	12	10.58
	444	0.205	1.899	0.84	0.000	0.380	1.761	−0.020	0.89	0.000	0.077	2.291	−0.604	0.89	0.000	27	19	12.18
	445	−0.043	1.673	0.53	0.003	0.007	1.630	−0.005	0.59	0.003	−0.064	1.740	−0.108	0.55	0.008	30	1	5.08
	446	−0.014	0.769	0.19	0.331	0.130	0.649	−0.015	0.84	0.000	−0.101	1.042	−0.431	0.67	0.000	29	9	2.22
	**Mean**	**0.073**	**1.082**	**0.59**	**0.090**	**0.025**	**1.123**	**0.000**	**0.81**	**0.001**	**0.094**	**1.014**	**0.100**	**0.75**	**0.010**	**29**	**NA**	**NA**
*q* = 2	**Mean**	0.021	0.760	0.50	0.138	−0.026	0.800	0.000	0.78	0.010	0.045	0.684	0.100	0.73	0.038	29	NA	0.00
*q* = 3	**Mean**	0.010	0.660	0.49	0.169	−0.038	0.701	0.000	0.78	0.006	0.036	0.576	0.100	0.73	0.034	29	NA	0.00
*q* = 4	**Mean**	0.005	0.614	0.48	0.190	−0.043	0.655	0.000	0.78	0.005	0.033	0.525	0.100	0.74	0.032	29	NA	0.00

^*^Only the *A*DTR models for *q* = 0 & 1 and the mean parameters for the whole cohort for *q* = 2–4 are listed here; to save page space the full results for *q* = 2–4 are presented in Suppl. Table S1A.

**Table 2:** The Beta-Diversity Time Relationship (*B*DTR) models for the individuals in the 32-healthy cohort*.

Order	Subject ID	Power law (PL)				PL with exponential cutoff (PLEC)					PL with inverse exponential cutoff (PLIEC)					*n*	PLEC Pred.	
		*w*	ln(*c*)	*R*	*p*-value	*w*	ln(*c)*	*d*	*R*	*p*-value	*w*	ln(*c*)	*d*	*R*	*p*-value		*A*_max_	*D*_max_
*q* = 0	400	0.542	−0.018	0.98	0.000	0.611	−0.075	−0.007	0.98	0.000	0.540	−0.010	−0.012	0.98	0.000	29	87	7.73
	401	0.450	0.162	0.98	0.000	0.506	0.114	−0.006	0.98	0.000	0.391	0.349	−0.297	0.98	0.000	30	84	6.37
	402	0.547	−0.267	0.98	0.000	0.342	−0.085	0.021	0.99	0.000	0.706	−0.772	0.812	0.99	0.000	31	NA	0.81
	403	0.454	−0.063	0.99	0.000	0.493	−0.098	−0.004	0.99	0.000	0.470	−0.113	0.081	0.99	0.000	32	123	5.94
	404	0.390	0.117	0.97	0.000	0.609	−0.071	−0.023	0.99	0.000	0.276	0.480	−0.577	0.98	0.000	30	26	3.73
	405	0.569	0.092	0.99	0.000	0.718	−0.040	−0.015	0.99	0.000	0.475	0.393	−0.484	0.99	0.000	31	48	7.53
	406	0.340	0.234	0.98	0.000	0.402	0.180	−0.006	0.98	0.000	0.240	0.553	−0.513	0.99	0.000	31	67	4.34
	407	0.425	0.161	0.99	0.000	0.548	0.061	−0.014	1.00	0.000	0.324	0.475	−0.489	1.00	0.000	28	39	4.58
	408	0.310	−0.013	0.99	0.000	0.237	0.048	0.008	0.99	0.000	0.339	−0.103	0.141	0.99	0.000	29	NA	1.36
	410	0.361	0.104	0.99	0.000	0.419	0.055	−0.006	0.99	0.000	0.306	0.275	−0.270	1.00	0.000	29	70	4.12
	411	0.293	0.153	0.98	0.000	0.352	0.104	−0.006	0.98	0.000	0.229	0.353	−0.314	0.98	0.000	29	59	3.27
	412	0.480	0.042	0.97	0.000	0.322	0.171	0.018	0.98	0.000	0.518	−0.074	0.182	0.98	0.000	28	NA	1.15
	413	0.324	0.060	0.96	0.000	0.491	−0.076	−0.019	0.98	0.000	0.237	0.330	−0.421	0.97	0.000	28	26	2.80
	414	0.310	0.050	0.98	0.000	0.403	−0.035	−0.009	0.99	0.000	0.272	0.170	−0.194	0.98	0.000	32	45	2.99
	415	0.415	0.270	0.97	0.000	0.635	0.080	−0.023	1.00	0.000	0.246	0.803	−0.849	0.99	0.000	30	28	4.72
	416	0.474	−0.031	1.00	0.000	0.522	−0.070	−0.005	1.00	0.000	0.467	−0.009	−0.034	1.00	0.000	28	104	6.26
	418	0.477	0.357	0.95	0.000	0.823	0.049	−0.035	0.99	0.000	0.234	1.129	−1.242	0.99	0.000	31	24	6.20
	420	0.490	−0.046	0.99	0.000	0.530	−0.079	−0.005	1.00	0.000	0.495	−0.062	0.024	0.99	0.000	28	106	6.44
	423	0.430	0.160	0.99	0.000	0.567	0.046	−0.015	1.00	0.000	0.333	0.465	−0.480	1.00	0.000	29	38	4.66
	424	0.465	0.105	0.96	0.000	0.730	−0.110	−0.029	0.99	0.000	0.331	0.522	−0.649	0.97	0.000	28	25	4.55
	429	0.434	0.063	0.99	0.000	0.486	0.020	−0.006	0.99	0.000	0.379	0.237	−0.275	0.99	0.000	29	81	5.31
	430	0.244	0.050	0.99	0.000	0.267	0.032	−0.003	0.99	0.000	0.216	0.134	−0.129	0.99	0.000	27	89	2.62
	431	0.267	0.116	0.98	0.000	0.265	0.118	0.000	0.98	0.000	0.224	0.248	−0.203	0.99	0.000	27	NA	NA
	432	0.488	0.194	0.98	0.000	0.713	0.006	−0.024	0.99	0.000	0.341	0.654	−0.724	0.99	0.000	29	30	5.53
	435	0.349	0.083	0.98	0.000	0.409	0.040	−0.007	0.98	0.000	0.277	0.299	−0.325	0.98	0.000	25	58	3.65
	436	0.222	0.203	0.97	0.000	0.305	0.128	−0.008	0.98	0.000	0.135	0.482	−0.453	0.99	0.000	32	38	2.54
	437	0.540	0.060	1.00	0.000	0.580	0.022	−0.004	1.00	0.000	0.510	0.155	−0.155	1.00	0.000	33	145	10.26
	439	0.281	−0.002	0.96	0.000	0.193	0.074	0.009	0.97	0.000	0.310	−0.094	0.146	0.96	0.000	30	NA	1.32
	443	0.493	−0.045	1.00	0.000	0.438	0.000	0.006	1.00	0.000	0.529	−0.157	0.175	1.00	0.000	28	NA	0.82
	444	0.308	0.110	0.99	0.000	0.348	0.079	−0.005	0.99	0.000	0.255	0.274	−0.253	1.00	0.000	27	70	3.35
	445	0.347	0.042	0.99	0.000	0.398	−0.001	−0.005	0.99	0.000	0.320	0.128	−0.136	0.99	0.000	30	80	3.83
	446	0.502	0.007	0.98	0.000	0.638	−0.108	−0.015	0.99	0.000	0.454	0.156	−0.235	0.98	0.000	29	43	5.19
	**Mean**	0.407	0.078	0.98	0.000	0.478	0.018	−0.008	0.99	0.000	0.356	0.240	−0.255	0.99	0.000	29	60	4.46
*q* = 1	**Mean**	0.108	0.073	0.76	0.024	0.140	0.047	−0.004	0.87	0.000	0.073	0.183	−0.171	0.87	0.001	29	35	1.50
*q* = 2	**Mean**	0.073	0.086	0.61	0.172	0.104	0.061	−0.004	0.79	0.032	0.033	0.209	−0.190	0.80	0.035	29	26	1.34
*q = 3*	**Mean**	0.074	0.091	0.63	0.098	0.105	0.067	−0.004	0.78	0.033	0.033	0.218	−0.196	0.80	0.030	29	26	1.36
*q = 4*	**Mean**	0.081	0.093	0.64	0.076	0.110	0.070	−0.003	0.78	0.023	0.040	0.218	−0.194	0.80	0.024	29	37	1.43

^*^Only the *B*DTR models for *q* = 0 and the mean parameters for the whole cohort for *q* = 1–4 are listed here; to save page space the full results for *q* = 2–4 are presented in Suppl. Table S1B.

#### Performance and parameter range of the DTR models

Regarding the alpha-diversity DTR or *A*DTR (Table 1 and Table S1A), the traditional PL model did perform universally well (*p*-values < 0.001) when *q* = 0, *i.e*., the traditional STR models. Although the PLEC and PLIEC performed slightly better than the PL model when *q* = 0, we select the PL model in consideration of the parsimony principle. However, systematic failures occurred when PL model was fitted to the higher order diversities (*q* = 1, 2, 3, 4). Instead, the two extended PL models, PLEC and PLIEC, especially the former, perform very well (*p* < 0.01) with the higher order *A*DTRs. Therefore, we conclude that, although traditional PL model is not applicable to the DTR at the higher diversity orders, either PLEC or PLIEC can be utilized to model the DTR for higher diversity orders (*q* = 1–4), with PLEC performing best. Between PLEC and PLIEC, we preferred PLEC because it not only allows for fluctuated diversity with time, but also can be utilized to predict maximal accumulation diversity.

As to the beta-diversity DTR or *B*DTR (Table 2 and Suppl. Table S1B), the performances of the three models (PL, PLEC, and PLIEC) are similar with the previous described *A*DTR modeling. Similar to the *A*DTR, the traditional PL model indeed encountered difficulties at higher order diversities, but its performance is generally better than it did with the *A*DTR. The PL model successfully fitted to the zero-order *B*DTR of all 32 subjects (*p* < 0.0001), and to the first-order *B*DTR of 32 subjects with an average *p*-value = 0.024. It indeed had some failures at the higher order (*q* = 2 to 4). As in the case of alpha-diversity, both PLEC and PLIEC fitted to the *B*DTR at all diversity orders significantly better than the traditional PL model. We therefore conclude that both PLEC and PLIEC can be utilized to describe the *B*DTR at higher diversity orders, with a preference to PLEC given that it can predict maximal accumulation diversity.

We adopted two modified PL models, PLEC and PLIEC, originally introduced to SAR modeling by Plotkin *et al*. and Ulrich & Buszko^52,53^, respectively (also see Tjørve)^13^. The former is termed *persistence function*-I and has maximum value, and the latter is termed *persistence function*-II. Essentially, PLEC and PLIEC can be considered as extensions to parameter *c*, rather than to *w*, *i.e*., $c(x)=c\,\exp \,(dx)$ or $c(x)=c\,\exp (d/x)$, respectively. Therefore, *w* is assumed to have the similar interpretation as its counterpart in the basic PL. PLEC and PLIEC, however, both behave very differently. The PLEC model asymptotically approaches *c****x***^*w*^ as ***x*** becomes small, whereas the PLIEC asymptotically approaches *c****x***^*w*^ as ***x*** becomes large. They were designed to remedy the potentially *unlimited accumulation* of species when the time approaches to infinity by introducing a taper-off exponent that may even produce asymptote. Indeed, both the models performed extremely well, usually outperforming the basic PL model, except in a handful case when the sample size was too small (3 or 4). As demonstrated previously, in the higher order diversity of the DTR, PLEC and PLIEC performed particularly well, while the basic PL failed.

The PLEC model has an advantage over the other two models in predicting the maximal diversity accumulation. In addition, we do concur with Plotkin *et al*. and Ulrich & Buszko^52,53^ that a taper-off parameter may indeed be necessary. The issue may not be serious for DAR, because both the Earth and the diversity on Earth are finite anyway. But time is potentially infinite; hence the traditional PL would predict an ever increasing (if *w* > 0) or ever decreasing (if *w* < 0) diversity, which could hardly be realistic. The issue can be particularly troubling in the case of human vaginal microbial communities because of the periodic nature of menses, which may cause periodic changes of the vaginal microbiota. From this perspective, the failure of traditional PL in modeling the DTR of vaginal microbiota should be anticipated; accordingly, the success of PLEC and PLIEC with taper-off effect should also be expected.

**Figure 1:**
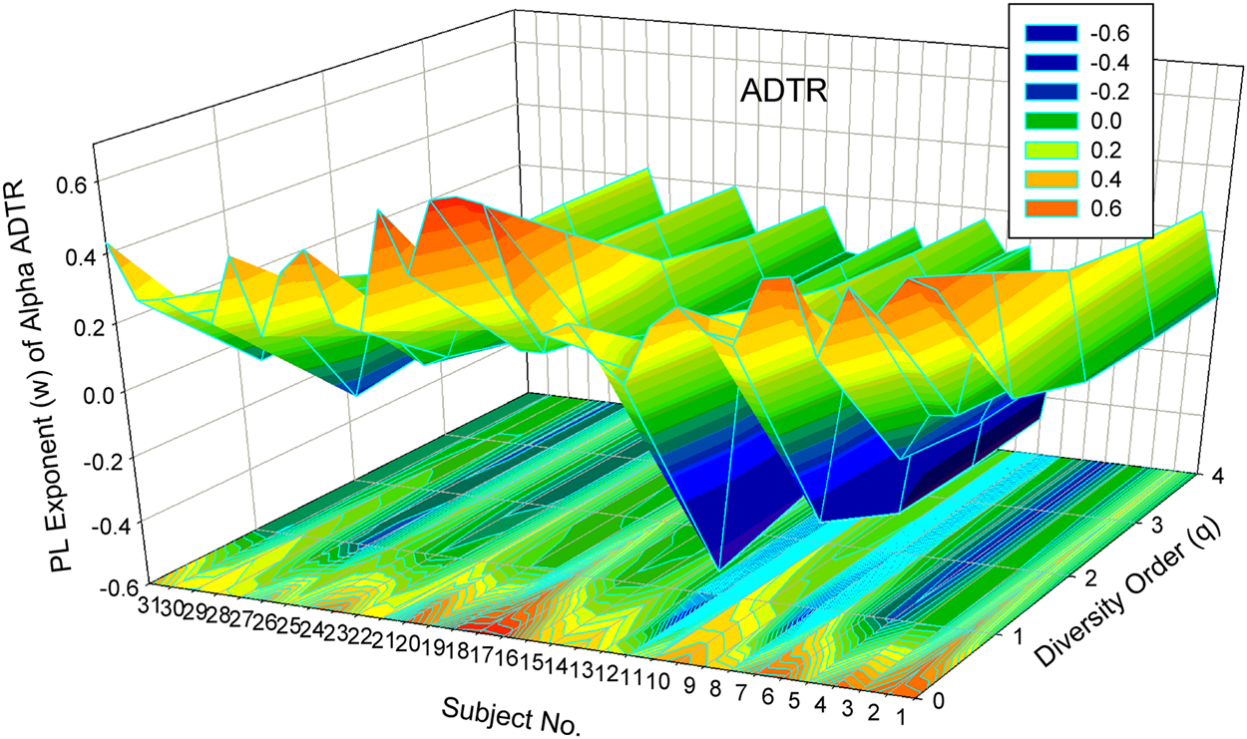
The 3D-graph of the power law exponent (*w*) of alpha*-*DTR.

The alpha DTR or *A*DTR has the average exponent *w* = (0.406, 0.073, 0.021, 0.010, 0.005) corresponding to the diversity order *q* = 0–4 (Table 1, Suppl. Table S1A); the beta DTR or *B*DTR has the average exponent *w* = (0.407, 0.018, 0.073, 0.074, 0.081), corresponding to the diversity order *q* = 0–4 (Table 2, Suppl. Table S1B). Note that the parameter (*w*) of *A*DTR displays a *monotonic decreasing relationship* with diversity order (*q*) (Fig. 1), and *w* of *B*DTR displays a *valley-shape relationship* (Fig. 3). It is also noted that *w* values listed above are the average of 32 individual subjects, and *w* for a specific individual subject may be negative.

It is also noted that the average PL exponent (*w*) of the zero-order *B*DTR model is equal to 0.407 for the HVM. This *w*-value is very close to its counterpart in the temporal scaling of alpha-diversity *A*DTR (*w* = 0.406), and both nonetheless have very different *ln*(*c*) values. The average *ln*(*c*) across all 32 subjects is 0.078 and 2.898 for *B*DTR and *A*DTR, respectively. Therefore, although the values of the scaling parameter (*w*) in both alpha- and beta-diversity are almost equal, the predicted diversities by the scaling models can be very different due to the significant difference in parameter *c*. The same magnitude of *w* in both alpha and beta DTR suggests that the rates of diversity accumulation at the zero-order (species-richness level or traditional STR) are nearly the same.

White *et al*.^61^ survey revealed that the temporal scaling exponent (*w*) is 0.10–0.51 with a mean of *w* = 0.29 through the comparison of 984 community type series of eukaryotic organisms. Oliver *et al*.^21^ survey of 72 bacteria STR found a range of 0.05–0.51 with a mean of *w* = 0.26. Shade *et al*.^22^ meta-analysis of 76 sites representing air, aquatic, soil, wastewater treatment, human- and plant-associated microbiomes, discovered a consistent STR with *w* ranging from 0.24 to 0.61. Our finding of *w* = 0.406 of *A*DTR with *q* = 0 for the species richness of HVM, which corresponds to the traditional STR, does fall in the ranges reported in the literature. Since our study, to the best of our knowledge, is the first investigation of the DTR beyond richness level (*q* = 0), we establish the first report on the alpha *A*DTR ranges for diversity order *q* = 1–4 as 0*.005–0.073* for the HVMs. Similarly, since we are not aware of any report on the beta- diversity scaling of *B*DTR, we establish the zero-order *B*DTR of the HVM as 0.407, and the range of the *B*TDR for order *q* = 1–4 as 0.018–0.081.

The possible negative exponent (*w*) in PL and its extended versions (PLEC & PLIEC) reveal an important difference between DAR (Ma)^49^ and DTR: the spatial scaling is usually positive, but the temporal scaling can be negative. That is, *diversity could decrease with time*, which makes sense even intuitively.

#### Visualization of the DTR models

**Figure 2:**
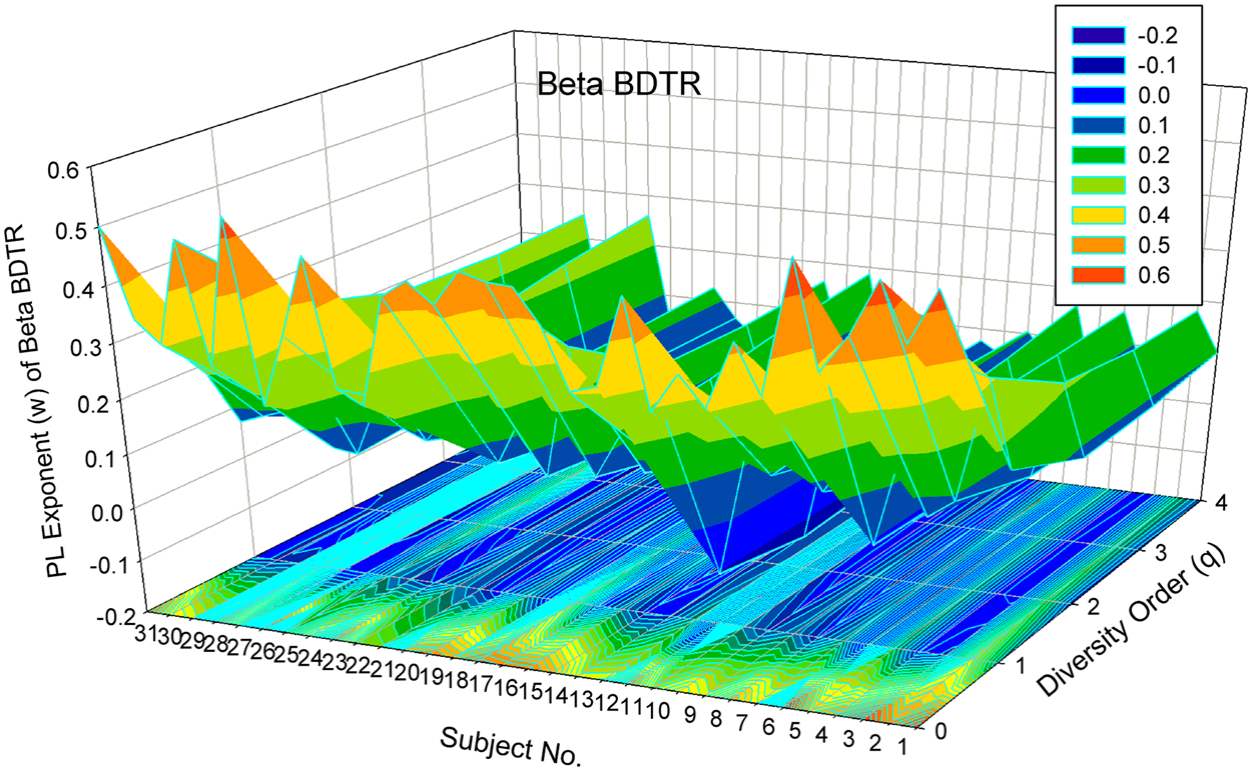
The 3D-graph of the power law exponent (*w*) of beta*-*DTR.

**Figure 3:**
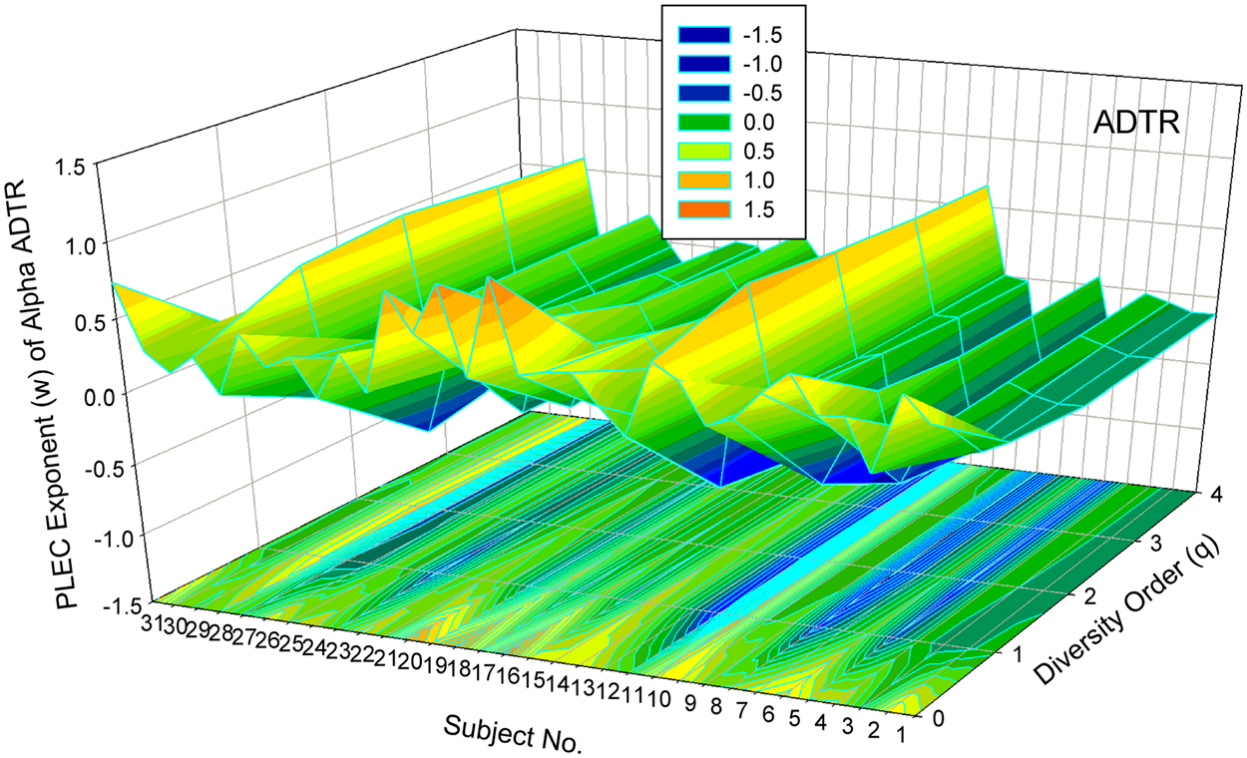
The 3D-graph of the PLEC exponent (*w*) of alpha alpha*-*DTR.

**Figure 4:**
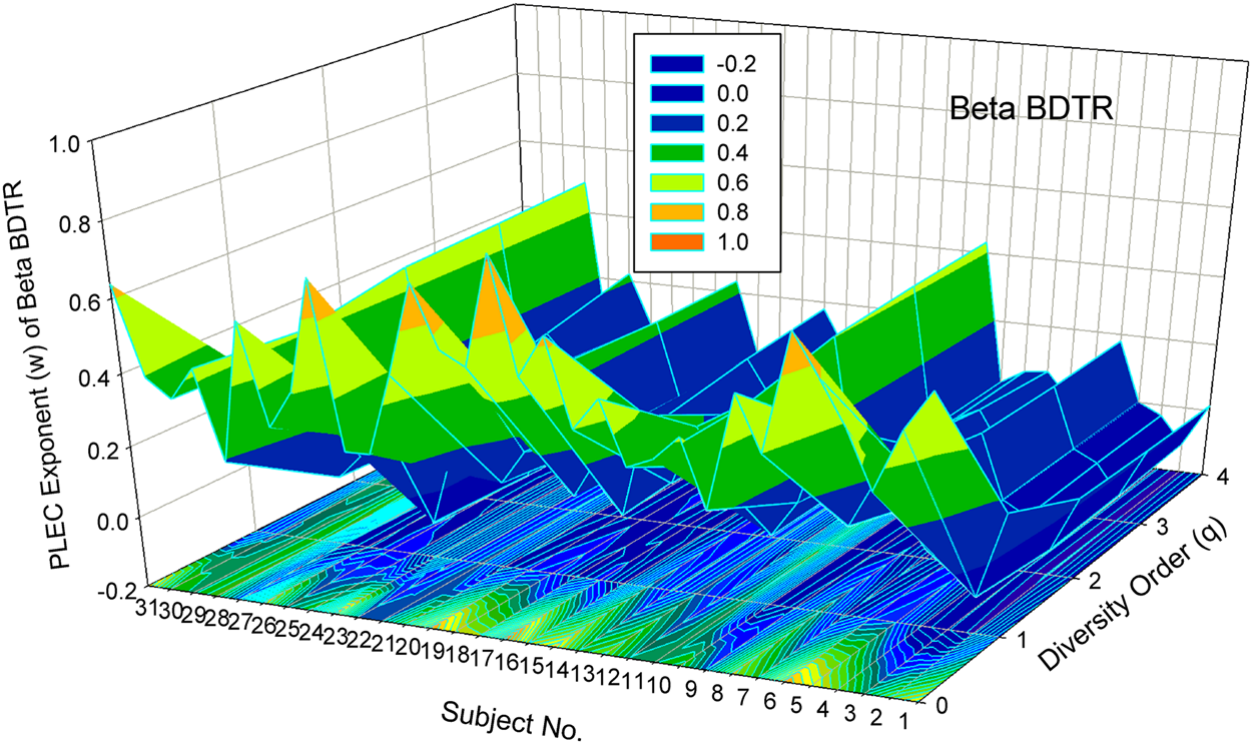
The 3D-graph of the PLEC exponent (*w*) of beta beta*-*DTR.

**Figure 5:**
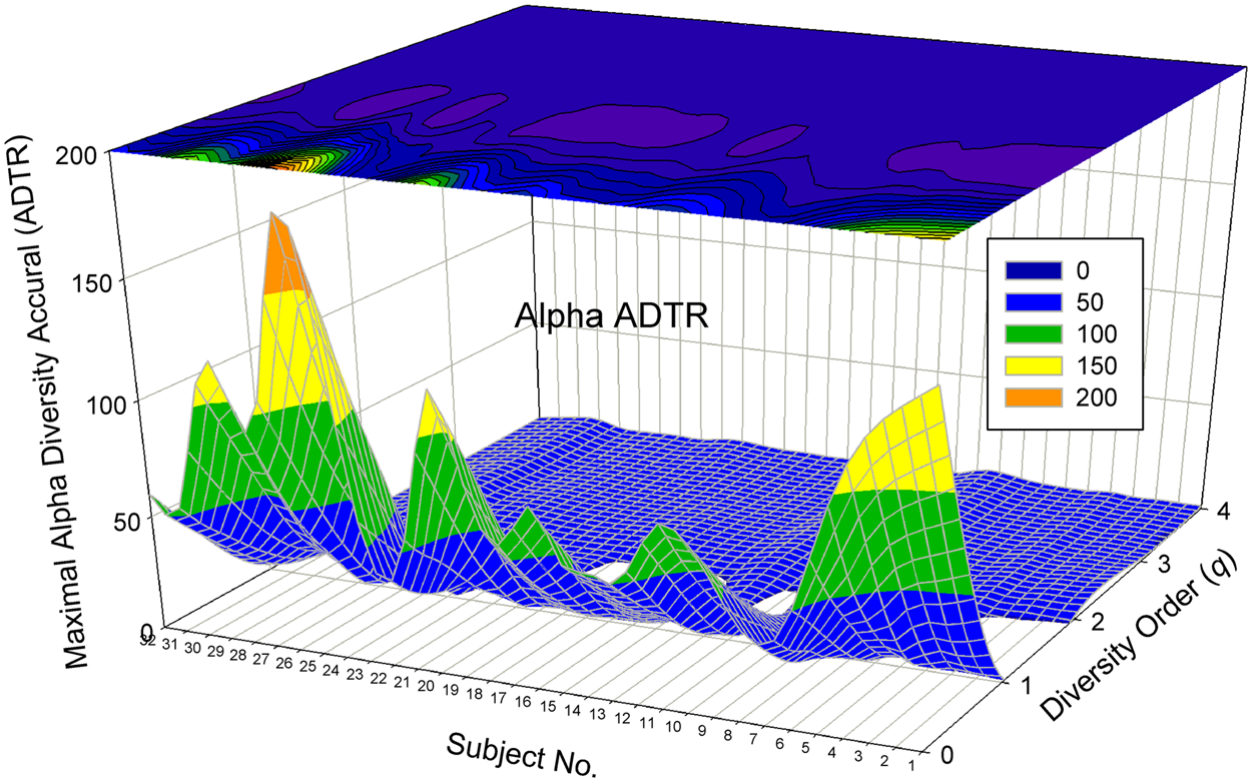
The 3D-graph of the alpha-MAD predicted with the PLEC model for alpha-DTR.

**Figure 6:**
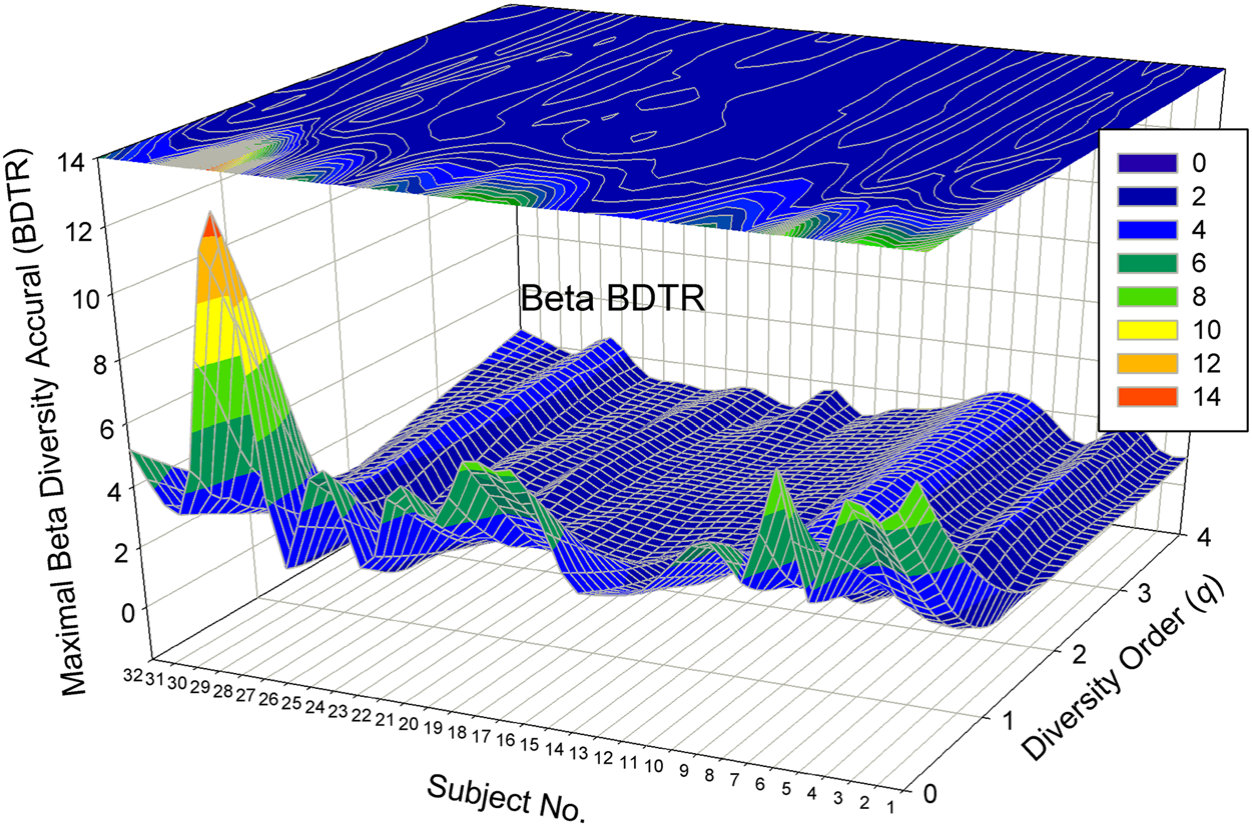
The 3D-graph of the beta-MAD predicted with the PLEC model for beta-DTR.

Figure 1 shows the 3D-graph of the PL exponent (*w*) of 32 alpha-DTR or *A*DTR models for 32 subjects at each diversity orders (*q* = 0–4), and Fig. 2 shows the beta-diversity counterpart, *i.e*., PL *w* of *B*DTR. Figures 3 and 4 show the PLEC counterparts, *i.e*., PLEC *w* of *A*DTR and PLEC *w* of *B*DTR, respectively. Figure 5 shows the 3D-graph alpha-MAD or *maximal accumulation* alpha *diversity*, predicted by the *A*DTR models for each of the 32 subjects at each diversity order (*q* = 0–4), and Fig. 6 shows the beta-diversity counterpart, *i.e*., beta-MAD or *maximal accumulation* beta *diversity*, predicted by the *B*DTR models.

Both Figs 1 and 2 show that negative PL *w*-values exist widely in DTR; especially with alpha diversity-time scaling (*A*DTR), *w* was drawn within the range between [–0.523 and + 0.656] (Fig. 1), a potential equilibrium around *w* = 0 (no change in diversity). With beta-diversity scaling (*B*DTR), *w* was drawn in the range between [−0.137 and +0.569] (Fig. 2), and the negative amplitude of accumulation (fluctuation) is smaller than the positive amplitude. Figures 1 and 2 also exhibit rather strong heterogeneities of *w* among 32 individual subjects, and the heterogeneity appears more significant at lower order diversities (especially for *q* = 0, *i.e*., the traditional STR).

The PLEC exponents (*w*) show similar pattern ranging from negative to positive, with the PLEC alpha *w* of *A*DTR ranging [0.935, 1.045], and the PLEC beta *w* of *B*DTR ranging [−0.125, 0.823]. As demonstrated previously, PLEC is more appropriate for DTR modeling since PL may fail to describe the DTR at higher diversity orders. One observation is that the range of alpha-diversity scaling exponent (*w*) is largely symmetrical around zero, but that of beta-diversity *w* is asymmetrical around zero, with a larger positive fluctuation.

The predicted maximal accumulation diversities or MADs (Figs 5 and 6) demonstrate high heterogeneity, varying significantly from individual to individual, especially at lower diversity orders. The alpha-MAD across individuals and diversity orders is 170 (Fig. 5), and the beta counterpart is 10.26 (Fig. 6). The latter predicts that the maximal beta diversity fluctuation in the 32-healthy cohort can be equivalent to 10 distinctly different communities according to the interpretation of multiplicative beta-diversity (Chao *et al*.)^40,41^.

#### The heterogeneities of the DTR parameters among individual subjects

The heterogeneities of the PL parameters [*w*, ln(*c*)] for DTR and those of the PLEC & PLIEC parameters [*w*, ln(*c*), *d*] were also captured quantitatively by fitting these parameters to two standard statistical distributions, *i.e*., the normal distribution and power law distribution [Eqn. (13)], which are often utilized to represent the typical symmetrical and asymmetrical heterogeneities—respectively. In Suppl. Table S3, we listed the distribution-fitting results, *i.e*., the *p*-values for testing the statistical distribution of each DTR parameter [*w*, ln(*c*), *d*] for five diversity orders (*q* = 0–4) and both diversity types (alpha and beta). Approximately 60% parameter cases followed the normal distribution and 97% cases followed the power law distribution. However, the predicted maximal accumulation diversities in all but one case (subject) followed the power law distribution. This suggests that, while the distributions of some parameters are symmetrical, the distributions of most parameters are highly skewed. Furthermore, the distribution of the maximal accumulation diversities among individual subjects is again highly skewed. It is noted that the statistical distribution here describes the distribution of the DTR model parameters among different individuals (communities) in the 32-healthy cohort, and hence the parameter (*K*) of the power law distribution depicts the asymmetricity (skewness) of the DTR parameters among individuals.

### Diversity-Time-Area Relationship (DTAR) modeling

The 32-healthy cohort dataset includes a total of approximately 900 HVM samples from 32 individuals, and 800 of them (excluding approximately 100 to allow for proper alignment of area-time units) were utilized to test the bivariate power law (BPL) DTAR model [Eqns (11, 12)], and the full results were exhibited in Suppl. Table S2A and B. To avoid the potential bias from arbitrarily ordering the sequence of individual subjects in accruing individuals (area) and corresponding diversities, 50 runs of random samplings from the total permutations of the 32 subjects were conducted to build 50 DTAR models separately, and the average parameters from the 50 runs were adopted as the final DTAR model parameters. The average BPL model parameters from Suppl. Tables S2A and B are summarized in the following Table 3. From Table 3 and Suppl. Tables 2A and B (detailed results from the 50 runs), we report the following findings as explained below.

**Table 3:** The Bivariate Power Law (BPL) parameters for the Diversity Time Area Relationship (DTAR) for the 32-healthy cohort*.

Diversity Order	BPL for Alpha Diversity Scaling						BPL for Beta Diversity Scaling					*n*
	*w-time*	z-area	*ln(c)*	*R*	*p*-value	*n*	*w*	z-area	ln(*c*)	*R*	*p*-value	
*q* = *0*	0.242	0.461	3.452	0.96	0.000	800	−0.151	0.404	0.598	0.98	0.000	775
*q* = *1*	−0.010	0.292	1.480	0.75	0.000	800	−0.100	0.240	0.674	0.92	0.000	775
*q* = *2*	−0.029	0.209	1.059	0.68	0.009	800	−0.076	0.201	0.674	0.81	0.000	775
*q* = *3*	−0.029	0.183	0.920	0.66	0.001	800	−0.078	0.205	0.689	0.77	0.000	775
*q* = *4*	−0.029	0.171	0.857	0.65	0.001	800	−0.083	0.217	0.701	0.77	0.000	775

*The results are based on the averages of 50 repetitions, and the full results for all repetitions, are supplied in Suppl. Tables S2A and B.

#### The performance of BPL in modeling the alpha DTAR and its parameter ranges

Regarding the alpha-diversity scaling of DTAR, the BPL [Eqns (11), (12)] fits to the DTAR data of the 32-healthy cohort highly significant with an average *p*-value = 0.01 across 50 runs (all but one has *p* < 0.001). The average temporal-scaling parameter *w* is (0.242, −0.010, −0.029, −0.029, −0.029) for *q* = 0^th^-order to 4^th^-order of diversities, respectively, exhibiting a decline trend with the increase of the diversity order (*q*) in general, becoming stabilized after order (*q* > 1), and negative scaling (*w*) beyond the zero-order diversity scaling (species richness). The average spatial-scaling parameter *z* is (0.461, 0.292, 0.209, 0.183, 0.171) for *q* = 0^th^-order to 4^th^-order of diversities, respectively, exhibiting a decline trend with the increase of the diversity order (*q*), but keeping all positive values.

The approximately twice size of the spatial-scaling parameter (*z*) over temporal-scaling parameter (*w*) at the diversity order *q* = 0, suggests that, although both have positive scaling effects at the species-richness level (0^th^-order Hill numbers), space (‘area’ or individual) is more significant than time in accumulating diversity (species). This certainly makes sense since it simply says that the inter-subject heterogeneity (variability) is more significant than the temporal heterogeneity (variability) of an individual subject in contributing the accumulation of species-richness across the members of a cohort. For the higher order diversities, the difference is even more categorical, with time having negative scaling effects at higher order beyond the basic species-richness level. This finding casts doubt on Preston^1^ ergodic conjecture; that is, space and time at least in ecological time, do not seem to be equivalent in terms of the accumulation of diversity.

#### The performance of BPL in modeling the beta DTAR and its parameter ranges

Regarding the beta-diversity scaling of DTAR, the same BPL, utilized for describing the alpha-diversity DTAR as described above, also fits to the beta-diversity DTAR of the 32-healthy cohort dataset extremely significant with all *p-*values < 0.001 for 250 tested models. The average temporal-scaling parameter *w* is (−0.151, −0.100, −0.076, −0.078, −0.083) for *q* = 0^th^-order to 4^th^-order of diversities, respectively, exhibiting a mostly increasing trend with the increase of the diversity order (*q*) but all negative (even for species richness) scaling *w*. This mostly increasing trend of temporal beta-diversity scaling is also opposite with the trend demonstrated by the alpha-diversity scaling summarized above. The average spatial-scaling parameter *z* of DTAR model is (0.404, 0.240, 0.201, 0.205, 0.217) for *q* = 0^th^-order to 4^th^-order of beta-diversities, respectively. The scaling trend exhibits a mixed non-monotonic trend (valley-shaped) with the increase of the diversity order (*q*). Note that all scaling parameters are positive with the spatial-scaling parameter (*z*). The results from the beta-diversity DTAR further suggest that space and time can be different in terms of the accumulation of beta-diversity; that is, Preston^1^ conjecture may not be true at the ecological time scale we tested. Indeed, the results in beta-diversity scaling indicate that even at species-richness level, time has a negative scaling parameter, and is different from space (area) in shaping the species accumulation.

**Figure 7:**
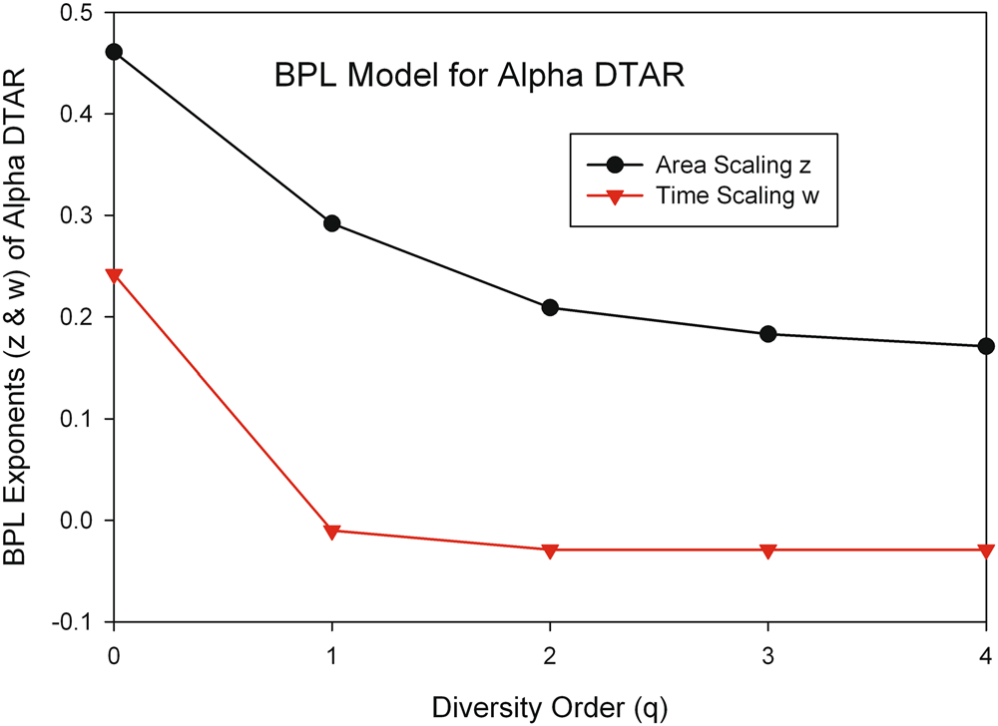
The parameters *z* & *w* of the BPL (bivariate power law) for the alpha-DTAR.

**Figure 8:**
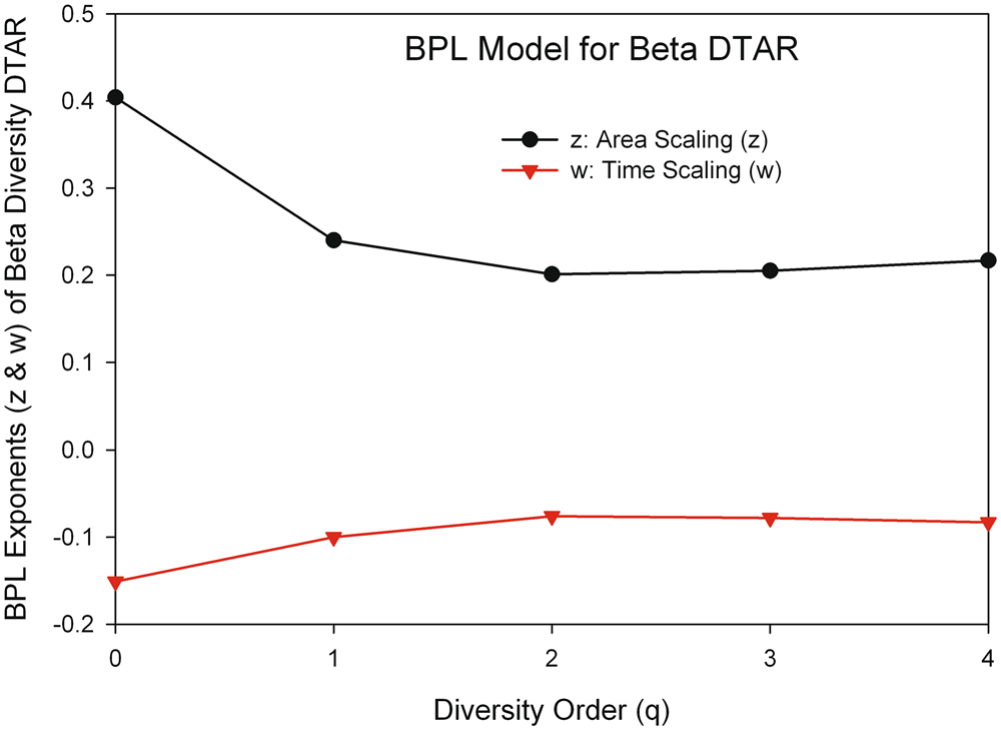
The parameters *z* & *w* of the BPL (bivariate power law) for the beta-DTAR.

Figure 7 shows the graph of the average BPL exponents (*z* & *w*) of the alpha DTAR models across 50 runs (each run produced a DTAR model) for each of the 5 diversity orders (*q* = 0–4). Figure 8 shows their beta-diversity counterparts. The above-described properties such as the ranges of BPL parameters (*z* & *w*) as well as their change trends with diversity order (*q*) all can be visually checked in Figs 7 and 8.

#### The distribution of DTAR parameters

Suppl. Table S4 demonstrates that the power law statistical distribution fits to the BPL parameters *z*, *w*, & *ln*(*c*) extremely significant, although the normal distribution also succeeded in 15 out of 60 cases, mostly for PLIEC or higher order beta-diversities (*q* = 2–4).

In conclusion, the scaling effects of both area and time can be adequately captured by the BPL, with *area* seemingly having a larger effect than time on diversity accumulation. *Area* seems to always affect diversity accumulation positively (*z* > 0). In contrast, beyond the species-richness level, *time* may have a negative effect on diversity accumulation (*w* may be less than *0*). Especially with the beta-diversity scaling, the effect of time can be negative even for species-richness accumulation. Therefore, Preston’s^1^ conjecture may not be true beyond species richness at the ecological time scale. It is generally recognized that the bacteria diversity of HVM is likely to experience periodic decline and restoration due to menses (Ma *et al*.)^36^. Therefore, a negatively temporal-scaling exponent is certainly possible. In addition, we suggest investigating the general DTAR further when more extensive joint spatial-temporal data sets are available.

## Discussion

Multiple mechanisms have been proposed to explain the STRs, including random sampling process at short time scales and ecological processes such as climatic variability, succession changes, meta-population dynamics at longer time scales (Drakare *et al*., Tjørve, Triantis *et al*., White *et al*., Williams *et al*.)^11,13,14,19,50,61,62^. However, direct experimental evidence to support these hypotheses is still scarce. Here, we only discuss one possible mechanism—the self-similarity or scale invariance, determined by the mathematical function of the DTR, *i.e*., the power law (Ma)^63^. Since the self-similarity property has been extensively studied in the context of the SAR, the following discussion aims to extend the results from the SAR to the STR and DTR.

From the basic power-law of the DTR, Eqn. (5), inspired by Harte *et al*.^64,65^, Sizling & Storch *et al*.^66^, Drakare *et al*.^14^, we can make the following derivations:

17 $$dD/dT=wD/T.$$

The exponent *w* is here the *proportionality constant* in the rate-equation form of the power law. The left side is the diversity increase per increase in time (unit), *i.e*., the tangent of the PL curve [Eqn. (5)], the right side is the *w* multiplied by *the diversity per unit time*, termed *temporal diversity density*. The above equation can be rewritten as the following equation:

18 $$\frac{dD/D}{dT/T}=w,$$

which further reveals that *w* is the ratio of diversity accumulation rate to time-increase rate, or the *relative diversity-accumulate rate*.

The second parameter (*c*) is *not* independent of the temporal sampling unit used, which makes the comparison of *c* between different case studies difficult. According to the self-similarity hypothesis, *c* is *the diversity in one unit of time*, that is, *D*_0_ = *c* when *T* = 1. It is the diversity within one unit of time, but *not* per unit of time.

Self-similarity is also known as scale-invariant, which refers to the following mathematical property of the power law:

19 $$f(\alpha T)=c{(\alpha T)}^{w}={\alpha }^{w}f(T)\propto f(T)$$

that is, scaling the argument *T* (time) by a constant factor *α*, is equivalent to scaling its function proportionally by a constant factor ${\alpha }^{w}.$ Therefore, all power laws with a particular scaling exponent *w* are equivalent up to constant factors because each is a scaled version of the others. The scale-invariance is also responsible for the linear relationship after log-transformation of power law [$\mathrm{ln}(D)=\,\mathrm{ln}(c)+w\,\mathrm{ln}(T)$], and the resulted straight line on log-log plot is termed the signature of power law. This is another reason we adopted log-log linear transformation fitting of the power law; in fact, the parameter *w* fitted with the linear transformation is the slope of the straight line, or the derivative of the equation [Eqn.(8)], which is independent of the parameter *c*. On the other hand, the slope or derivative of untransformed PL function [Eqn.(5)] is dependent on both *w* and *c*, rather than on *w* only as in the transformed fitting [Eqn. (8)].

From (19), it is also obvious that:

20 $${D}_{\alpha T}/{D}_{T}={\alpha }^{w}$$

Applying log function with the base α on both sides of (20), there is:

21 $${\mathrm{log}}_{\alpha }({D}_{\alpha T}/{D}_{T})={\mathrm{log}}_{\alpha }{\alpha }^{w}=w\,{\mathrm{log}}_{\alpha }\alpha =w$$

It follows that

22 $$D=c{T}^{{\mathrm{log}}_{\alpha }({D}_{\alpha T}/{D}_{T})}$$

If *α* = 2, then $$w={\mathrm{log}}_{2}({D}_{2T}/D{}_{T})$$

23 $$D=c{T}^{{\mathrm{log}}_{2}({D}_{2T}/{D}_{T})}$$

is a special case of (22).

Inspired by Tjørve *et al*.^12^, we can further derive the following relationship between *diversity overlap* and *w*-value. The fraction (*h*) of new diversity due to expansion of α times of the original time *T* can be expressed as:

24 $$h=({D}_{\alpha T}-{D}_{T})/{D}_{T}={\alpha }^{w}-1$$

Similarly, the proportion of new diversity in the *j*-th time (of the same length) added can be computed with the following equation:

25 $${h}_{j}=({D}_{jT}-{D}_{(j-1)T})/{D}_{T}={j}^{w}-{(j-1)}^{w}$$

Similar to Tjørve *et al*.^12^, we term *α* as *time multiplication rate*, and *h* is the fraction of new diversity accumulated as a function of *w*. When α = 2, the proportion of new diversity $h={2}^{w}-1$, the diversity overlap (*g*) of two bordering times of the same length (computed as the proportion of the new diversity in the second time window) is as:

26 $$g=(2{D}_{T}-{D}_{2T})/{D}_{T}=2-{2}^{w}$$

In (26), *g* is also the scale-invariant overlap because it is the overlap between two time windows of the same duration. Equation (26) can be utilized to estimate the *diversity overlap* between two bordering time windows with the same duration (length). If *w* = 1, then *g* = 0, no overlap; and if *w* = 0, *g* = 1, totally overlap. Detailed discussion on these equations can be found in Tjørve *et al*.^12^, where comparisons with Harte *et al*.^64,65^, Sizling & Storch^66^, as well as the limitations of these derivations were also discussed.

Oliver *et al*.^21^ emphasized a key future importance of studying the microbial STR is to distinguish between anthropogenic perturbations and underlying natural dynamics, and to offer ecological insights for clinical benefit in fighting bacterial infections. The test of the STR (extended to DTR in our study) in the human microbiome, besides establishing its validity in the arguably the closest and the most important ecological communities to our body, does offer additional benefits. One potential clinical application of DTR is the previously mentioned suggestion made by Oliver *et al*.^21^ to distinguish between anthropogenic perturbations and underlying natural dynamics and to offer ecological insights for clinical benefit in fighting bacterial infections. The DTR models as well as the formulae derived above based on self-similarity property can be particular useful in the case of human vaginal microbial communities in assessing or predicting the bacterial temporal turnover of HVM associated with menses. In gut microbiome, the emerging medical practice aimed to restore gut microbial diversity by fecal microbiota transplantation (Borody *et al*., O’Doherty *et al*.)^67,68^ should also require assessment of diversity across space (individual) and/or time, in which the DTR (DTAR) may also play a critical role.

Finally, we would like to remind readers that DTAR scaling is far more complex. Especially the beta-diversity scaling with DTAR suggests that space and time can be different even at the species-richness level. This suggests that Preston^1^ conjecture may not be true at the ecological time scale we tested. Time (period) and space (area) are most likely different in shaping the diversity accumulation in general. Future mechanistic studies on DTR/DTAR in microbiome ecology, similar to those performed by macro-ecologists such as Alder *et al*., Ulrich, and McGlinn & Palmer^3–7^, are urgently needed to further this important field.

## Electronic supplementary material

Supplementary Information (I)

Supplementary Information (II)
