# Supplementary material for: Diversity time-period and diversity-time-area relationships exemplified by the human microbiome
Source: Sci Rep. 2018 May 8;8:7214. doi: 10.1038/s41598-018-24881-3 (PMC5940795; doi:10.1038/s41598-018-24881-3)
Supplement: Supplementary file 1 — Supplementary Information (I) [file 41598_2018_24881_MOESM1_ESM.pdf]

Supplementary Information (I) to: Ma ZS (2017) Diversity time-period and diversity-time-area relationships exemplified by the human microbiome

List of Supplementary Information

The following materials are included in the online Supporting Information (I), i.e., this MS-Word document.
Supplementary information to "Test Results" Section, "Diversity Time Relationship (DTR) modeling" Sub-section:
Suppl. Table S1A. Alpha Diversity Time Relationship (*A*DTR) with 32 Healthy Cohort Dataset
Suppl. Table S1B. Beta-Diversity Time Relationship (*B*DTR) with 32 Healthy Cohort Dataset

The following materials are included in the online Supporting Information (II), i.e., the additional MS-Excel file.
Supplementary information to "Test Results" Section, "Diversity-Time-Area Relationship (DTAR) modeling" Sub-section:
Suppl. Table S2A. Alpha-Diversity Time Area Relationship (A-DTAR) with 32-Healthy Cohort Data
Suppl. Table S2B. Beta Diversity Time Area Relationship (B-DTAR) with 32-Healthy Cohort Data
Suppl. Table S3. The *p*-value of the Distribution-fitting for the parameters of DTR models
Suppl. Table S4. The *p*-value of the Distribution-fitting for the parameters of DTAR models

Supplementary information to Section "Test Results", Sub-section "Diversity Time-Period Relationship (DTR) modeling"

We utilized the HVM (human vaginal microbiome) datasets from the 32-healthy cohort study (Gajer et al. 2012) to test the DTR models. We construct three DTR models (PL, PLEC, and PLIEC) for each of the 32 subjects, with the time-series data of their vaginal microbial communities, respectively. A total of 960 DTR models were built. To save page space in the main text, Tables 1A and 1B in the main text only listed the partial results of alpha *A*DTR (for q=0, 1) and beta *B*DTR (for q=0), respectively. Suppl. Tables S1A and S1B below report the full results for diversity order q=0-4. A brief summary on the findings from these tables is presented in the main text. A relatively detailed discussion on the findings is presented here.

(*i*) *The performance of three DTR models* (*PL, PLEC & PLIEC*)
The model fittings are statistically extremely significant with zero-order alpha *A*DTR, *i.e*., the traditional STR, and all 96 models (3 models * 32 subjects) have *p*-values< 0.001 (Suppl. Table S1A). However, the results with higher order (q) alpha diversities are mixed. With the first-order *A*DTR, *i.e*., the temporal scaling of Shannon diversity index, only 27 out of the 32 subjects (85%) fit to the traditional PL model (*p*-value=0.1), and as high as 15% subjects fail to fit to the PL. With the 2nd, 3rd, and 4th order diversity, the traditional PL model failed to fit to most of the 32 subjects, and the failure rates were as high as 90%. We therefore, consider that the traditional PL is only applicable to the zero-order diversity, and not applicable to the high-order DTRs.

However, the two extended PL models, PLEC and PLIEC, especially the former, perform very well with the higher order *A*DTRs. Among the 32 subjects, PLEC successfully fitted to all 32 (average *p*-value=0.001), 31 (average p=0.01), 31 (average *p*-value=0.006), and 31 (average p=0.005) subjects, from the first to fourth order (*q=1 to 4*), respectively. With PLIEC model, among the 32 subjects, the number of significantly fitted models is 30 (average p=0.01), 26 (average p=0.038), 29 (average p=0.034), and 29 (average *p*-value=0.032) from the first to fourth order (q=1-4), respectively. Therefore, we conclude that, although traditional PL model is not applicable to the DTR at the higher diversity orders, either PLEC or PLIEC can be utilized to model the DTR, with PLEC performing best.

As to the beta-diversity *B*DTR (Suppl. Table S1B), the performances of the three models (PL, PLEC, and PLIEC) are similar with the previous described *A*DTR modeling. Similar to *A*DTR, the traditional PL model indeed encountered difficulties at higher order diversities, but its performance is generally better than it did with *A*DTR. The PL successfully fitted to the zero-order *B*DTR of all 32 subjects (p<0.0001), and to the first-order *B*DTR of 32 subjects with an average *p*-value=0.024. It indeed had some failures at the higher order (q=2 to 4). As in the case of alpha-diversity, both PLEC and PLIEC fitted to the *B*DTR at all diversity orders significantly well: the PLEC model succeeded in 90 out of 96 cases (32 subjects * 3 models) (p<0.05) and failed in 6 cases, and the PLIEC succeeded in 92 out of 96 cases (p<0.05).

In conclusion, the traditional PL model is only generally applicable at the zero-order DTR, *i.e.,* the traditional STR model. The two alternatives models PLEC and PLIEC instead performed equally well with all five q-orders, even when the traditional PL model often failed at the higher order (q=1-4). Between PLEC and PLIEC for DTR, we prefer the former because it has a potential maximal and an asymptote, which is desirable in describing possibly fluctuating DTR.

(*ii*) *The parameter range of the alpha ADTR models*

Regarding the alpha *A*DTR scaling, the average PL exponent (*w*) of zero-order ADTR model (*i.e*., traditional STR) is equal to 0.406 for the HVM. With the increase of the diversity order, *w* not only decreases, but also displays negative values; the number of negatives *vs*. positive exponent is 8/24, 11/21, 13/19, 13/19 from the 1st to 4th order, respectively.

The average PLEC exponent (*w*) of *A*DTR is 0.441 for zero-order diversity, and it further decrease with the increase of the diversity order (*q*), with a series (averages across 32 subjects) of 0.025, -0.132, -0.038, and -0.043 corresponding to the 1st, 2nd, 3rd and 4th order diversities, respectively. There are also negative exponents with PLEC, and the ratio of negative *vs*. positive exponent is 14/18, 17/15, 17/15, 18/14 for the 1st to 4th order, respectively.

The *average* PLIEC exponent (*w*) of *A*DTR is 0.379 for zero-order diversity, and it further decreases with the increase of the diversity order (*q*), with a series (averages across 32 subjects) of 0.094, 0.045, 0.036, and 0.033 corresponding to the 1st, 2nd, 3rd and 4th order diversities, respectively. The ratio of negative *vs*. positive exponent is 9/23, 12/20, 13/19, 12/20 for the 1st to 4th order, respectively.

The possible negative exponent (*w*) in PL and its extended versions (PLEC & PLIEC) reveal an important difference between DAR and DTR: the spatial scaling is always positive, but the temporal scaling can be negative. That is, *diversity could decrease with time,* which makes sense even intuitively.

Another important observation is the sign of the parameter *d* of PLEC and PLIEC models (Suppl. Table S1A & S1B) displayed both negative and positive values in the DTR modeling of temporal scaling. Interestingly, it is noted that in many cases, positive *d*-values concurred with negative *w*-values, but the opposite symbol matching also abounds in our results (Suppl. Table S1A). In the latter cases, usually positive *d*-values are paired with relatively smaller *w*-values than those concurred with negative *d*-values. The phenomenon raises a serious question: are the observed negative *w*-values in PLEC and PLIEC a statistical artifact from the model fitting (possibly due to the taper-off effect of the parameter *d*)*,* or a biologically meaningful mechanism? The fact that even PL model, which does not have a third parameter *d,* also display negative *w*-values, suggests that the observed pattern of *w* and *d* should primarily be attributed to some biological mechanisms, rather than statistical artifact or random effects.

(*iii*) *The parameter range of the beta BDTR models*
Regarding the temporal scaling of beta-diversity BDTR, the average PL exponent (*w*) of the zero-order *B*DTR model is equal to 0.407 for the HVM. This *w*-value is very close to its counterpart in the temporal scaling of alpha-diversity *A*DTR (*w*=0.406), and both nonetheless have very different *ln*(*c*) values. The average *ln*(*c*) across all 32 subjects is 0.078 and 2.898 for *B*DTR and *A*DTR, respectively. Therefore, although the values of the scaling parameter (*w*) in both alpha and beta-diversity are almost equal, the predicted diversities by the scaling models can be very different due to the significant difference in parameter *c*. The same magnitude of *w* in both alpha and beta DTR suggests that the rates of diversity accrual at the zero-order (species richness level or traditional STR), are nearly the same.

With the increase of the diversity order (*q*), the PL exponent (*w*) of *B*DTR decreases with the diversity order: the average *w-values* across 32 subjects equal 0.407, 0.108, 0.073, 0.074, 0.081 for order *q*=0, 1, 2, 3, 4, respectively. Furthermore, both positive and negative values occurred and the ratio of negative *vs*. positive exponents is 0/32, 2/30, 7/25, 8/24, 8/24 for order *q*=0, 1, 2, 3, 4, respectively.

Since PL model is not sufficient for describing the higher order *B*DTR, we need to invoke PLEC or PLIEC to describe the *B*DTR. The average PLEC exponent (*w*) across 32 individuals from 0 to 4th order is 0.478, 0.140, 0.104, 0.105, and 0.110, decreasing with the rising order (*q*). The ratio of negative *vs*. positive exponent is 0/32, 3/28, 5/27, 6/26, and 6/26 from the 0 to 4th order, respectively. Furthermore, the average taper-off parameter *d* is -0.008, -0.004, -0.004, -0.004, -0.003 from 0 to 4th order, respectively; the ratio of negative *vs*. positive taper-off parameter *d* is 26/6, 17/5, 17/5, 17/5, 17/5 from the 0 to 4th order, respectively.

The average series of the PLIEC exponent (*w*) of *B*DTR across 32 subjects is 0.356, 0.073, 0.033, 0.033, and 0.040, decreasing with the rising diversity order of *q=0,…,4*, respectively. The ratio of negative *vs*. positive exponent is 0/32, 10/32, 11/32, 12/32, and 12/32 corresponding to the rising *q*-order. Furthermore, for the rising *q*, the average taper-off parameter *d* is -0.255, -0.171, -0.190, -0.196, -0.194, respectively; the corresponding ratio of negative *vs*. positive taper-off parameter *d* is 25/7, 20/12, 19/13, 17/15, and 18/14. The performance and behavior of PLEC and PLIEC are similar, but the magnitudes of *d* in both the models are very different, as expected.

Table S1A. Alpha Diversity Time Relationship (ADTR) with 32-Healthy Cohort HVM Dataset

**Order**	**Subject ID**	**Power law (PL)**				**PL with exponential cutoff (PLEC)**					**PL with inverse exponential cutoff (PLIEC)**					***n***	**PLEC Pred.**	
		w	**ln(*c*)**	***R***	***p*-value**	***w***	**ln(*c*)**	***d***	***R***	***p*-value**	***w***	**ln(*c*)**	***d***	***R***	***p*-value**		A_{max}	D_{max}
q = 0	400	0.542	2.312	0.96	0.000	0.440	2.397	0.011	0.96	0.000	0.705	1.800	0.805	0.97	0.000	29	NA	7
	401	0.565	2.669	0.93	0.000	0.608	2.632	-0.005	0.93	0.000	0.394	3.207	-0.856	0.94	0.000	30	122	140
	402	0.463	2.567	0.92	0.000	0.083	2.904	0.039	0.97	0.000	0.736	1.697	1.398	0.96	0.000	31	NA	17
	403	0.527	1.670	0.96	0.000	0.422	1.765	0.010	0.97	0.000	0.636	1.321	0.566	0.97	0.000	32	NA	5
	404	0.329	3.358	0.98	0.000	0.409	3.289	-0.008	0.98	0.000	0.266	3.558	-0.318	0.98	0.000	30	51	89
	405	0.537	2.075	0.96	0.000	0.649	1.975	-0.011	0.96	0.000	0.470	2.287	-0.341	0.96	0.000	31	59	53
	406	0.523	2.585	0.96	0.000	0.507	2.599	0.002	0.96	0.000	0.434	2.866	-0.451	0.96	0.000	31	NA	NA
	407	0.337	3.635	0.99	0.000	0.322	3.647	0.002	0.99	0.000	0.346	3.608	0.042	0.99	0.000	28	NA	76
	408	0.366	2.702	0.98	0.000	0.258	2.792	0.012	0.98	0.000	0.385	2.643	0.093	0.98	0.000	29	NA	19
	410	0.428	2.810	0.98	0.000	0.524	2.729	-0.010	0.98	0.000	0.300	3.209	-0.629	0.99	0.000	29	52	72
	411	0.366	3.282	0.91	0.000	0.623	3.066	-0.028	0.95	0.000	0.093	4.136	-1.345	0.98	0.000	29	22	80
	412	0.198	2.854	0.96	0.000	0.131	2.909	0.007	0.97	0.000	0.206	2.828	0.040	0.96	0.000	28	NA	22
	413	0.261	3.015	0.99	0.000	0.304	2.980	-0.005	1.00	0.000	0.235	3.095	-0.126	0.99	0.000	28	61	51
	414	0.349	2.861	0.96	0.000	0.495	2.729	-0.014	0.98	0.000	0.273	3.108	-0.401	0.97	0.000	32	35	55
	415	0.310	3.069	0.94	0.000	0.476	2.927	-0.017	0.96	0.000	0.157	3.554	-0.772	0.97	0.000	30	28	57
	416	0.403	2.776	0.98	0.000	0.437	2.749	-0.004	0.98	0.000	0.329	3.006	-0.358	0.99	0.000	28	109	79
	418	0.643	2.187	0.95	0.000	1.045	1.831	-0.041	0.99	0.000	0.337	3.161	-1.566	0.99	0.000	31	25	65
	420	0.656	1.946	0.97	0.000	0.380	2.169	0.031	0.99	0.000	0.865	1.297	1.011	0.99	0.000	28	NA	6
	423	0.640	2.590	0.97	0.000	0.965	2.318	-0.035	0.99	0.000	0.397	3.351	-1.198	0.99	0.000	29	28	95
	424	0.434	3.448	0.95	0.000	0.627	3.292	-0.021	0.97	0.000	0.330	3.770	-0.502	0.96	0.000	28	30	121
	429	0.602	2.401	0.97	0.000	0.885	2.163	-0.031	0.99	0.000	0.411	2.997	-0.940	0.99	0.000	29	29	70
	430	0.259	3.533	0.98	0.000	0.204	3.576	0.006	0.99	0.000	0.327	3.324	0.322	0.99	0.000	27	NA	48
	431	0.274	3.194	0.96	0.000	0.439	3.064	-0.019	0.99	0.000	0.170	3.513	-0.491	0.98	0.000	27	23	55
	432	0.472	2.783	0.94	0.000	0.127	3.072	0.037	0.99	0.000	0.715	2.022	1.198	0.98	0.000	29	NA	20
	435	0.400	2.861	0.98	0.000	0.377	2.878	0.003	0.98	0.000	0.461	2.678	0.276	0.99	0.000	25	NA	28
	436	0.212	4.450	0.98	0.000	0.291	4.379	-0.008	0.99	0.000	0.156	4.630	-0.292	0.99	0.000	32	36	170
	437	0.432	3.020	0.95	0.000	0.491	2.965	-0.006	0.95	0.000	0.473	2.885	0.221	0.95	0.000	33	82	103
	439	0.232	2.924	0.91	0.000	0.069	3.064	0.017	0.95	0.000	0.325	2.628	0.472	0.94	0.000	30	NA	21
	443	0.253	3.905	0.99	0.000	0.300	3.868	-0.005	0.99	0.000	0.222	4.000	-0.147	0.99	0.000	28	60	121
	444	0.285	3.703	0.98	0.000	0.175	3.790	0.013	0.99	0.000	0.372	3.436	0.411	0.99	0.000	27	NA	50
	445	0.274	2.765	0.98	0.000	0.298	2.745	-0.002	0.98	0.000	0.288	2.720	0.071	0.98	0.000	30	149	51
	446	0.431	2.793	0.88	0.000	0.740	2.534	-0.033	0.92	0.000	0.326	3.122	-0.519	0.89	0.000	29	22	60
	**Mean**	**0.406**	**2.898**	**0.96**	**0.000**	**0.441**	**2.869**	**0.000**	**0.97**	**0.000**	**0.379**	**2.983**	**-0.100**	**0.97**	**0.000**	**29**	**NA**	**NA**
q=1	400	0.107	0.582	0.54	0.002	-0.032	0.698	0.015	0.62	0.002	0.277	0.049	0.839	0.71	0.000	29	2	2.03
	401	0.450	0.104	0.83	0.000	-0.117	0.593	0.060	0.94	0.000	0.781	-0.942	1.663	0.89	0.000	30	2	1.88
	402	0.040	0.874	0.32	0.080	-0.161	1.052	0.020	0.76	0.000	0.128	0.592	0.453	0.51	0.015	31	8	2.40

	403	0.033	0.234	0.43	0.015	-0.033	0.295	0.007	0.56	0.004	0.061	0.147	0.142	0.47	0.027	32	5	1.32
	404	-0.107	1.922	0.52	0.003	-0.397	2.172	0.030	0.79	0.000	0.010	1.552	0.590	0.60	0.002	30	13	4.68
	405	-0.024	0.362	0.15	0.408	0.203	0.161	-0.023	0.65	0.000	-0.203	0.934	-0.919	0.67	0.000	31	9	1.49
	406	0.232	0.517	0.73	0.000	-0.117	0.826	0.036	0.86	0.000	0.474	-0.253	1.237	0.84	0.000	31	3	2.24
	407	-0.326	2.532	0.85	0.000	-0.472	2.650	0.016	0.86	0.000	-0.302	2.457	0.117	0.85	0.000	28	30	4.59
	408	0.161	1.478	0.96	0.000	0.141	1.495	0.002	0.96	0.000	0.159	1.483	-0.009	0.96	0.000	29	NA	6.38
	410	0.202	0.161	0.88	0.000	0.075	0.267	0.014	0.91	0.000	0.242	0.034	0.200	0.88	0.000	29	NA	1.34
	411	0.274	1.454	0.67	0.000	0.813	1.003	-0.058	0.87	0.000	-0.231	3.036	-2.492	0.94	0.000	29	14	10.35
	412	-0.523	2.040	0.98	0.000	-0.585	2.090	0.007	0.98	0.000	-0.550	2.126	-0.133	0.98	0.000	28	84	1.09
	413	0.019	1.646	0.17	0.394	-0.204	1.827	0.025	0.83	0.000	0.144	1.257	0.605	0.60	0.004	28	8	4.97
	414	0.142	1.372	0.95	0.000	0.195	1.324	-0.005	0.96	0.000	0.105	1.491	-0.193	0.96	0.000	32	39	6.32
	415	0.070	0.727	0.33	0.074	0.395	0.447	-0.034	0.73	0.000	-0.107	1.286	-0.890	0.57	0.005	30	12	2.78
	416	0.046	0.620	0.42	0.027	-0.162	0.788	0.023	0.89	0.000	0.157	0.275	0.537	0.68	0.000	28	7	1.88
	418	0.134	0.201	0.72	0.000	0.302	0.051	-0.017	0.82	0.000	0.033	0.522	-0.516	0.78	0.000	31	18	1.86
	420	0.441	-0.255	0.77	0.000	-0.150	0.224	0.066	0.88	0.000	0.884	-1.630	2.141	0.87	0.000	28	2	1.29
	423	0.082	0.719	0.31	0.098	0.440	0.420	-0.039	0.66	0.001	-0.282	1.857	-1.792	0.82	0.000	29	11	2.85
	424	0.081	1.048	0.46	0.014	-0.230	1.300	0.035	0.87	0.000	0.325	0.290	1.181	0.87	0.000	28	7	3.00
	429	0.134	0.072	0.91	0.000	0.145	0.064	-0.001	0.91	0.000	0.169	-0.036	0.170	0.92	0.000	29	145	1.90
	430	0.224	1.941	0.88	0.000	0.286	1.892	-0.007	0.88	0.000	0.277	1.777	0.253	0.88	0.000	27	41	14.40
	431	0.036	1.908	0.42	0.030	0.114	1.847	-0.009	0.56	0.010	0.022	1.951	-0.065	0.43	0.089	27	13	7.56
	432	0.002	0.638	0.01	0.966	-0.450	1.017	0.049	0.77	0.000	0.165	0.129	0.802	0.35	0.174	29	9	1.60
	435	0.083	0.973	0.66	0.000	0.051	0.996	0.004	0.67	0.001	0.068	1.016	-0.065	0.67	0.002	25	NA	2.89
	436	0.144	2.444	0.85	0.000	0.157	2.432	-0.001	0.85	0.000	0.200	2.264	0.293	0.87	0.000	32	157	21.52
	437	-0.120	0.790	0.76	0.000	-0.260	0.921	0.013	0.85	0.000	0.033	0.296	0.811	0.93	0.000	33	20	1.49
	439	-0.020	1.375	0.15	0.444	-0.292	1.610	0.029	0.85	0.000	0.158	0.815	0.892	0.72	0.000	30	10	3.41
	443	0.166	1.796	0.58	0.001	0.614	1.433	-0.050	0.87	0.000	-0.097	2.612	-1.272	0.75	0.000	28	12	10.58
	444	0.205	1.899	0.84	0.000	0.380	1.761	-0.020	0.89	0.000	0.077	2.291	-0.604	0.89	0.000	27	19	12.18
	445	-0.043	1.673	0.53	0.003	0.007	1.630	-0.005	0.59	0.003	-0.064	1.740	-0.108	0.55	0.008	30	1	5.08
	446	-0.014	0.769	0.19	0.331	0.130	0.649	-0.015	0.84	0.000	-0.101	1.042	-0.431	0.67	0.000	29	9	2.22
	**Mean**	**0.073**	**1.082**	**0.59**	**0.090**	**0.025**	**1.123**	**0.000**	**0.81**	**0.001**	**0.094**	**1.014**	**0.100**	**0.75**	**0.010**	**29**	**NA**	**NA**
$q = 2$	400	0.008	0.376	0.08	0.680	-0.136	0.497	0.016	0.58	0.005	0.156	-0.086	0.727	0.76	0.000	29	9	1.41
	401	0.328	-0.064	0.83	0.000	-0.140	0.339	0.049	0.97	0.000	0.605	-0.939	1.392	0.91	0.000	30	3	1.39
	402	0.000	0.522	0.00	0.996	-0.099	0.610	0.010	0.59	0.002	0.027	0.436	0.139	0.21	0.525	31	10	1.62
	403	0.017	0.117	0.34	0.056	-0.031	0.160	0.005	0.53	0.009	0.036	0.057	0.098	0.40	0.082	32	6	1.14
	404	-0.155	1.538	0.59	0.001	-0.528	1.859	0.039	0.84	0.000	0.027	0.963	0.915	0.70	0.000	30	14	2.75
	405	-0.026	0.195	0.27	0.137	0.101	0.083	-0.013	0.62	0.001	-0.136	0.544	-0.560	0.68	0.000	31	8	1.21
	406	0.090	0.378	0.41	0.023	-0.278	0.704	0.038	0.82	0.000	0.350	-0.449	1.330	0.77	0.000	31	7	1.54
	407	-0.469	2.170	0.84	0.000	-0.838	2.470	0.041	0.89	0.000	-0.312	1.686	0.755	0.86	0.000	28	20	2.18
	408	0.133	1.163	0.93	0.000	0.068	1.218	0.007	0.95	0.000	0.153	1.102	0.097	0.93	0.000	29	NA	3.60
	410	0.096	0.027	0.85	0.000	0.015	0.095	0.009	0.90	0.000	0.130	-0.079	0.167	0.87	0.000	29	NA	1.09

	411	0.209	1.008	0.61	0.000	0.734	0.569	-0.057	0.89	0.000	-0.230	2.382	-2.165	0.93	0.000	29	13	5.53
	412	-0.456	1.508	0.95	0.000	-0.719	1.721	0.029	0.98	0.000	-0.292	1.000	0.792	0.97	0.000	28	25	1.14
	413	-0.020	1.258	0.17	0.387	-0.217	1.419	0.022	0.74	0.000	0.089	0.921	0.526	0.53	0.016	28	10	3.12
	414	0.132	0.981	0.95	0.000	0.163	0.953	-0.003	0.95	0.000	0.119	1.024	-0.070	0.95	0.000	32	54	4.23
	415	0.013	0.416	0.09	0.635	0.260	0.202	-0.026	0.76	0.000	-0.135	0.882	-0.741	0.59	0.003	30	10	1.72
	416	0.010	0.335	0.19	0.323	-0.098	0.423	0.012	0.88	0.000	0.068	0.155	0.280	0.62	0.002	28	8	1.37
	418	0.029	0.135	0.38	0.036	0.115	0.058	-0.009	0.61	0.001	-0.041	0.358	-0.359	0.63	0.001	31	13	1.27
	420	0.233	-0.189	0.76	0.000	-0.069	0.056	0.034	0.86	0.000	0.465	-0.909	1.122	0.86	0.000	28	2	1.08
	423	0.029	0.406	0.18	0.345	0.237	0.232	-0.022	0.59	0.004	-0.183	1.068	-1.042	0.75	0.000	29	11	1.75
	424	0.045	0.564	0.30	0.123	-0.255	0.808	0.033	0.88	0.000	0.285	-0.179	1.158	0.89	0.000	28	8	1.72
	429	0.061	0.008	0.89	0.000	0.057	0.011	0.000	0.89	0.000	0.084	-0.064	0.113	0.91	0.000	29	NA	NA
	430	0.224	1.461	0.86	0.000	0.181	1.495	0.005	0.87	0.000	0.366	1.026	0.671	0.91	0.000	27	NA	6.00
	431	-0.037	1.532	0.31	0.116	-0.009	1.510	-0.003	0.32	0.262	0.017	1.366	0.256	0.39	0.139	27	NA	4.52
	432	-0.057	0.481	0.31	0.100	-0.307	0.690	0.027	0.66	0.001	-0.014	0.346	0.212	0.34	0.210	29	11	1.28
	435	0.041	0.502	0.42	0.036	-0.031	0.555	0.009	0.52	0.031	0.050	0.476	0.041	0.42	0.112	25	3	1.73
	436	0.214	1.481	0.93	0.000	0.199	1.495	0.002	0.93	0.000	0.296	1.219	0.426	0.95	0.000	32	NA	7.40
	437	-0.072	0.350	0.76	0.000	-0.173	0.445	0.010	0.89	0.000	0.032	0.016	0.547	0.98	0.000	33	17	1.13
	439	-0.077	0.984	0.50	0.005	-0.352	1.221	0.029	0.90	0.000	0.115	0.378	0.964	0.84	0.000	30	12	2.00
	443	0.084	1.426	0.26	0.178	0.686	0.938	-0.067	0.83	0.000	-0.306	2.635	-1.884	0.70	0.000	28	10	6.35
	444	0.101	1.484	0.48	0.012	0.456	1.205	-0.041	0.85	0.000	-0.176	2.335	-1.311	0.84	0.000	27	11	6.34
	445	-0.024	1.264	0.24	0.205	0.083	1.172	-0.011	0.51	0.019	-0.081	1.442	-0.283	0.38	0.116	30	8	3.51
	446	-0.030	0.488	0.44	0.018	0.103	0.376	-0.014	0.92	0.000	-0.121	0.773	-0.449	0.83	0.000	29	7	1.61
	**Mean**	0.021	0.760	0.50	0.138	-0.026	0.800	0.000	0.78	0.010	0.045	0.684	0.100	0.73	0.038	29	NA	0.00
$q = 3$	400	-0.003	0.314	0.03	0.877	-0.132	0.423	0.014	0.62	0.002	0.129	-0.097	0.648	0.80	0.000	29	9	1.30
	401	0.281	-0.080	0.82	0.000	-0.134	0.278	0.044	0.97	0.000	0.524	-0.848	1.222	0.91	0.000	30	3	1.30
	402	-0.004	0.419	0.06	0.745	-0.080	0.488	0.008	0.57	0.004	0.015	0.359	0.097	0.19	0.592	31	10	1.47
	403	0.014	0.091	0.33	0.065	-0.025	0.126	0.004	0.52	0.009	0.029	0.042	0.079	0.39	0.091	32	6	1.11
	404	-0.159	1.394	0.58	0.001	-0.556	1.735	0.042	0.85	0.000	0.048	0.739	1.042	0.72	0.000	30	13	2.35
	405	-0.022	0.155	0.28	0.123	0.078	0.066	-0.010	0.62	0.001	-0.109	0.432	-0.445	0.68	0.000	31	8	1.16
	406	0.054	0.350	0.28	0.125	-0.295	0.660	0.036	0.83	0.000	0.309	-0.460	1.304	0.79	0.000	31	8	1.40
	407	-0.479	2.007	0.84	0.000	-0.919	2.364	0.049	0.90	0.000	-0.265	1.342	1.035	0.86	0.000	28	19	1.80
	408	0.111	1.037	0.87	0.000	0.012	1.120	0.011	0.93	0.000	0.150	0.915	0.193	0.88	0.000	29	NA	3.03
	410	0.074	0.019	0.85	0.000	0.010	0.072	0.007	0.90	0.000	0.100	-0.064	0.131	0.86	0.000	29	NA	1.07
	411	0.182	0.871	0.59	0.001	0.683	0.452	-0.054	0.90	0.000	-0.220	2.131	-1.984	0.92	0.000	29	13	4.49
	412	-0.407	1.313	0.94	0.000	-0.699	1.550	0.033	0.98	0.000	-0.194	0.653	1.029	0.97	0.000	28	21	1.12
	413	-0.033	1.135	0.28	0.146	-0.213	1.281	0.020	0.70	0.000	0.067	0.824	0.486	0.53	0.015	28	11	2.69
	414	0.126	0.844	0.93	0.000	0.143	0.828	-0.002	0.93	0.000	0.127	0.841	0.006	0.93	0.000	32	72	3.65
	415	0.006	0.333	0.05	0.775	0.207	0.159	-0.021	0.77	0.000	-0.116	0.717	-0.612	0.60	0.002	30	10	1.53
	416	0.007	0.263	0.17	0.395	-0.078	0.331	0.009	0.88	0.000	0.052	0.123	0.218	0.61	0.003	28	9	1.27
	418	0.019	0.109	0.32	0.077	0.086	0.050	-0.007	0.57	0.004	-0.037	0.289	-0.289	0.61	0.001	31	12	1.20

	420	0.180	-0.147	0.76	0.000	-0.052	0.041	0.026	0.86	0.000	0.358	-0.701	0.863	0.85	0.000	28	2	1.06
	423	0.022	0.318	0.18	0.351	0.187	0.180	-0.018	0.58	0.004	-0.143	0.838	-0.818	0.74	0.000	29	10	1.54
	424	0.036	0.443	0.28	0.146	-0.218	0.649	0.028	0.88	0.000	0.239	-0.187	0.982	0.90	0.000	28	8	1.52
	429	0.047	0.005	0.89	0.000	0.044	0.008	0.000	0.89	0.000	0.065	-0.051	0.088	0.90	0.000	29	NA	NA
	430	0.208	1.278	0.85	0.000	0.102	1.362	0.012	0.87	0.000	0.389	0.721	0.859	0.93	0.000	27	NA	4.16
	431	-0.058	1.352	0.41	0.035	-0.055	1.350	0.000	0.41	0.114	0.032	1.076	0.426	0.52	0.021	27	NA	0.00
	432	-0.061	0.425	0.37	0.048	-0.268	0.598	0.022	0.64	0.001	-0.036	0.344	0.127	0.38	0.133	29	12	1.22
	435	0.033	0.389	0.40	0.046	-0.034	0.437	0.008	0.52	0.029	0.044	0.354	0.052	0.41	0.134	25	4	1.52
	436	0.227	1.214	0.96	0.000	0.205	1.235	0.002	0.96	0.000	0.296	0.994	0.358	0.98	0.000	32	NA	5.79
	437	-0.056	0.268	0.75	0.000	-0.136	0.343	0.008	0.89	0.000	0.026	0.006	0.431	0.98	0.000	33	17	1.10
	439	-0.090	0.858	0.58	0.001	-0.354	1.085	0.028	0.92	0.000	0.102	0.249	0.969	0.89	0.000	30	13	1.72
	443	0.043	1.299	0.14	0.482	0.630	0.824	-0.065	0.80	0.000	-0.352	2.525	-1.909	0.69	0.000	28	10	5.08
	444	0.043	1.360	0.21	0.285	0.431	1.056	-0.045	0.82	0.000	-0.270	2.323	-1.484	0.84	0.000	27	10	4.95
	445	-0.009	1.098	0.08	0.662	0.131	0.976	-0.015	0.54	0.009	-0.088	1.347	-0.397	0.40	0.097	30	9	3.09
	446	-0.028	0.401	0.45	0.014	0.091	0.301	-0.013	0.92	0.000	-0.111	0.660	-0.408	0.84	0.000	29	7	1.47
	**Mean**	0.010	0.660	0.49	0.169	-0.038	0.701	0.000	0.78	0.006	0.036	0.576	0.100	0.73	0.034	29	NA	0.00
$q = 4$	400	-0.004	0.285	0.05	0.781	-0.124	0.385	0.013	0.62	0.002	0.117	-0.095	0.598	0.81	0.000	29	10	1.26
	401	0.257	-0.080	0.82	0.000	-0.128	0.251	0.040	0.97	0.000	0.481	-0.789	1.127	0.91	0.000	30	3	1.26
	402	-0.004	0.375	0.07	0.707	-0.072	0.436	0.007	0.57	0.004	0.013	0.323	0.085	0.19	0.594	31	10	1.41
	403	0.012	0.081	0.33	0.066	-0.023	0.113	0.003	0.52	0.010	0.026	0.038	0.070	0.39	0.093	32	8	1.09
	404	-0.160	1.320	0.58	0.001	-0.562	1.667	0.042	0.85	0.000	0.058	0.634	1.092	0.73	0.000	30	13	2.16
	405	-0.020	0.138	0.28	0.122	0.069	0.059	-0.009	0.61	0.001	-0.098	0.386	-0.398	0.68	0.000	31	8	1.14
	406	0.040	0.338	0.22	0.228	-0.295	0.635	0.034	0.83	0.000	0.289	-0.455	1.275	0.80	0.000	31	9	1.34
	407	-0.474	1.918	0.83	0.000	-0.935	2.292	0.051	0.90	0.000	-0.236	1.180	1.150	0.86	0.000	28	18	1.66
	408	0.096	0.972	0.82	0.000	-0.019	1.068	0.012	0.92	0.000	0.146	0.814	0.248	0.85	0.000	29	2	2.94
	410	0.065	0.017	0.85	0.000	0.009	0.064	0.006	0.90	0.000	0.089	-0.057	0.116	0.86	0.000	29	NA	1.06
	411	0.168	0.811	0.57	0.001	0.657	0.402	-0.053	0.91	0.000	-0.217	2.015	-1.897	0.91	0.000	29	12	4.05
	412	-0.381	1.222	0.93	0.000	-0.678	1.462	0.033	0.97	0.000	-0.154	0.515	1.101	0.97	0.000	28	21	1.09
	413	-0.039	1.072	0.32	0.093	-0.212	1.212	0.019	0.68	0.000	0.059	0.767	0.475	0.54	0.013	28	11	2.49
	414	0.120	0.775	0.92	0.000	0.129	0.766	-0.001	0.92	0.000	0.130	0.745	0.049	0.92	0.000	32	129	3.54
	415	0.005	0.298	0.05	0.794	0.185	0.142	-0.019	0.77	0.000	-0.104	0.643	-0.549	0.60	0.002	30	10	1.46
	416	0.006	0.234	0.16	0.403	-0.069	0.295	0.008	0.88	0.000	0.046	0.110	0.194	0.61	0.003	28	9	1.24
	418	0.017	0.098	0.32	0.083	0.076	0.045	-0.006	0.57	0.005	-0.033	0.259	-0.258	0.61	0.002	31	13	1.18
	420	0.160	-0.131	0.76	0.000	-0.046	0.036	0.023	0.86	0.000	0.319	-0.623	0.767	0.85	0.000	28	2	1.05
	423	0.020	0.284	0.18	0.349	0.168	0.160	-0.016	0.58	0.004	-0.128	0.747	-0.730	0.74	0.000	29	11	1.47
	424	0.032	0.396	0.28	0.150	-0.197	0.581	0.026	0.88	0.000	0.216	-0.173	0.887	0.90	0.000	28	8	1.46
	429	0.042	0.004	0.89	0.000	0.039	0.007	0.000	0.89	0.000	0.058	-0.045	0.079	0.90	0.000	29	NA	NA
	430	0.196	1.185	0.83	0.000	0.056	1.295	0.016	0.87	0.000	0.397	0.567	0.953	0.95	0.000	27	NA	3.65
	431	-0.064	1.254	0.43	0.025	-0.075	1.263	0.001	0.43	0.084	0.041	0.930	0.500	0.57	0.009	27	75	2.76
	432	-0.060	0.395	0.39	0.039	-0.250	0.553	0.020	0.64	0.001	-0.039	0.328	0.105	0.39	0.113	29	13	1.19

435	0.030	0.346	0.40	0.048	-0.032	0.391	0.008	0.53	0.029	0.040	0.313	0.049	0.41	0.136	25	4	1.46
436	0.229	1.109	0.97	0.000	0.200	1.134	0.003	0.97	0.000	0.289	0.915	0.314	0.98	0.000	32	NA	4.77
437	-0.050	0.239	0.75	0.000	-0.121	0.305	0.007	0.89	0.000	0.023	0.005	0.384	0.98	0.000	33	17	1.08
439	-0.093	0.798	0.60	0.000	-0.349	1.019	0.027	0.93	0.000	0.100	0.189	0.969	0.91	0.000	30	13	1.61
443	0.027	1.226	0.09	0.647	0.586	0.773	-0.062	0.79	0.000	-0.357	2.418	-1.856	0.69	0.000	28	9	4.50
444	0.019	1.294	0.09	0.640	0.407	0.989	-0.045	0.81	0.000	-0.303	2.282	-1.524	0.85	0.000	27	9	4.39
445	-0.003	1.017	0.02	0.903	0.157	0.879	-0.017	0.58	0.004	-0.095	1.308	-0.463	0.43	0.061	30	9	2.92
446	-0.026	0.361	0.45	0.014	0.083	0.269	-0.012	0.92	0.000	-0.102	0.598	-0.374	0.84	0.000	29	7	1.41
**Mean**	0.005	0.614	0.48	0.190	-0.043	0.655	0.000	0.78	0.005	0.033	0.525	0.100	0.74	0.032	29	NA	NA

Table S1B. Beta Diversity Time Relationship (BDTR) with 32-Healthy Cohort HMV Dataset

Order	Subject ID	Power law (PL)				PL with exponential cutoff (PLEC)					PL with inverse exponential cutoff (PLIEC)					n	PLEC Pred.	
		w	ln(c)	R	p-value	w	ln(c)	d	R	p-value	w	ln(c)	d	R	p-value		A_{max}	D_{max}
	400	0.542	-0.018	0.98	0.000	0.611	-0.075	-0.007	0.98	0.000	0.540	-0.010	-0.012	0.98	0.000	29	87	7.73
	401	0.450	0.162	0.98	0.000	0.506	0.114	-0.006	0.98	0.000	0.391	0.349	-0.297	0.98	0.000	30	84	6.37
	402	0.547	-0.267	0.98	0.000	0.342	-0.085	0.021	0.99	0.000	0.706	-0.772	0.812	0.99	0.000	31	NA	0.81
	403	0.454	-0.063	0.99	0.000	0.493	-0.098	-0.004	0.99	0.000	0.470	-0.113	0.081	0.99	0.000	32	123	5.94
	404	0.390	0.117	0.97	0.000	0.609	-0.071	-0.023	0.99	0.000	0.276	0.480	-0.577	0.98	0.000	30	26	3.73
	405	0.569	0.092	0.99	0.000	0.718	-0.040	-0.015	0.99	0.000	0.475	0.393	-0.484	0.99	0.000	31	48	7.53
	406	0.340	0.234	0.98	0.000	0.402	0.180	-0.006	0.98	0.000	0.240	0.553	-0.513	0.99	0.000	31	67	4.34
	407	0.425	0.161	0.99	0.000	0.548	0.061	-0.014	1.00	0.000	0.324	0.475	-0.489	1.00	0.000	28	39	4.58
	408	0.310	-0.013	0.99	0.000	0.237	0.048	0.008	0.99	0.000	0.339	-0.103	0.141	0.99	0.000	29	NA	1.36
	410	0.361	0.104	0.99	0.000	0.419	0.055	-0.006	0.99	0.000	0.306	0.275	-0.270	1.00	0.000	29	70	4.12
	411	0.293	0.153	0.98	0.000	0.352	0.104	-0.006	0.98	0.000	0.229	0.353	-0.314	0.98	0.000	29	59	3.27
	412	0.480	0.042	0.97	0.000	0.322	0.171	0.018	0.98	0.000	0.518	-0.074	0.182	0.98	0.000	28	NA	1.15
	413	0.324	0.060	0.96	0.000	0.491	-0.076	-0.019	0.98	0.000	0.237	0.330	-0.421	0.97	0.000	28	26	2.80
	414	0.310	0.050	0.98	0.000	0.403	-0.035	-0.009	0.99	0.000	0.272	0.170	-0.194	0.98	0.000	32	45	2.99
	415	0.415	0.270	0.97	0.000	0.635	0.080	-0.023	1.00	0.000	0.246	0.803	-0.849	0.99	0.000	30	28	4.72
	416	0.474	-0.031	1.00	0.000	0.522	-0.070	-0.005	1.00	0.000	0.467	-0.009	-0.034	1.00	0.000	28	104	6.26
$q = 0$	418	0.477	0.357	0.95	0.000	0.823	0.049	-0.035	0.99	0.000	0.234	1.129	-1.242	0.99	0.000	31	24	6.20
	420	0.490	-0.046	0.99	0.000	0.530	-0.079	-0.005	1.00	0.000	0.495	-0.062	0.024	0.99	0.000	28	106	6.44
	423	0.430	0.160	0.99	0.000	0.567	0.046	-0.015	1.00	0.000	0.333	0.465	-0.480	1.00	0.000	29	38	4.66
	424	0.465	0.105	0.96	0.000	0.730	-0.110	-0.029	0.99	0.000	0.331	0.522	-0.649	0.97	0.000	28	25	4.55
	429	0.434	0.063	0.99	0.000	0.486	0.020	-0.006	0.99	0.000	0.379	0.237	-0.275	0.99	0.000	29	81	5.31
	430	0.244	0.050	0.99	0.000	0.267	0.032	-0.003	0.99	0.000	0.216	0.134	-0.129	0.99	0.000	27	89	2.62
	431	0.267	0.116	0.98	0.000	0.265	0.118	0.000	0.98	0.000	0.224	0.248	-0.203	0.99	0.000	27	NA	NA
	432	0.488	0.194	0.98	0.000	0.713	0.006	-0.024	0.99	0.000	0.341	0.654	-0.724	0.99	0.000	29	30	5.53
	435	0.349	0.083	0.98	0.000	0.409	0.040	-0.007	0.98	0.000	0.277	0.299	-0.325	0.98	0.000	25	58	3.65
	436	0.222	0.203	0.97	0.000	0.305	0.128	-0.008	0.98	0.000	0.135	0.482	-0.453	0.99	0.000	32	38	2.54
	437	0.540	0.060	1.00	0.000	0.580	0.022	-0.004	1.00	0.000	0.510	0.155	-0.155	1.00	0.000	33	145	10.26
	439	0.281	-0.002	0.96	0.000	0.193	0.074	0.009	0.97	0.000	0.310	-0.094	0.146	0.96	0.000	30	NA	1.32
	443	0.493	-0.045	1.00	0.000	0.438	0.000	0.006	1.00	0.000	0.529	-0.157	0.175	1.00	0.000	28	NA	0.82
	444	0.308	0.110	0.99	0.000	0.348	0.079	-0.005	0.99	0.000	0.255	0.274	-0.253	1.00	0.000	27	70	3.35
	445	0.347	0.042	0.99	0.000	0.398	-0.001	-0.005	0.99	0.000	0.320	0.128	-0.136	0.99	0.000	30	80	3.83
	446	0.502	0.007	0.98	0.000	0.638	-0.108	-0.015	0.99	0.000	0.454	0.156	-0.235	0.98	0.000	29	43	5.19
	**Mean**	0.407	0.078	0.98	0.000	0.478	0.018	-0.008	0.99	0.000	0.356	0.240	-0.255	0.99	0.000	29	60	4.46
$q = 1$	400	0.156	-0.061	0.87	0.000	0.148	-0.054	0.001	0.87	0.000	0.183	-0.144	0.131	0.87	0.000	29	NA	1.53
	401	0.310	-0.379	0.82	0.000	-0.095	-0.030	0.042	0.94	0.000	0.589	-1.260	1.403	0.92	0.000	30	2	0.99

	402	0.088	-0.043	0.80	0.000	-0.033	0.065	0.012	0.92	0.000	0.142	-0.214	0.276	0.84	0.000	31	3	1.07
	403	0.054	-0.030	0.89	0.000	0.020	0.001	0.003	0.92	0.000	0.078	-0.106	0.123	0.92	0.000	32	NA	1.02
	404	0.183	0.061	0.90	0.000	0.239	0.013	-0.006	0.91	0.000	0.156	0.148	-0.138	0.91	0.000	30	40	1.92
	405	-0.010	0.217	0.08	0.687	0.193	0.037	-0.021	0.68	0.000	-0.170	0.728	-0.821	0.70	0.000	31	9	1.31
	406	0.153	0.008	0.85	0.000	0.055	0.095	0.010	0.88	0.000	0.198	-0.134	0.228	0.86	0.000	31	NA	1.13
	407	0.075	0.452	0.46	0.015	0.322	0.252	-0.027	0.77	0.000	-0.177	1.236	-1.222	0.93	0.000	28	12	2.07
	408	0.133	-0.023	0.95	0.000	0.102	0.003	0.003	0.96	0.000	0.160	-0.107	0.132	0.96	0.000	29	NA	1.23
	410	0.093	-0.082	0.86	0.000	0.001	-0.006	0.010	0.93	0.000	0.153	-0.271	0.297	0.91	0.000	29	NA	0.99
	411	0.135	0.370	0.71	0.000	0.356	0.185	-0.024	0.86	0.000	-0.045	0.936	-0.892	0.88	0.000	29	15	2.20
	412	-0.103	0.626	0.48	0.010	0.177	0.398	-0.031	0.73	0.000	-0.432	1.644	-1.587	0.94	0.000	28	6	1.70
	413	0.160	0.060	0.86	0.000	0.276	-0.035	-0.013	0.90	0.000	0.084	0.294	-0.365	0.88	0.000	28	21	1.70
	414	0.081	0.006	0.95	0.000	0.110	-0.020	-0.003	0.96	0.000	0.072	0.037	-0.051	0.95	0.000	32	37	1.31
	415	0.070	0.189	0.50	0.005	0.290	-0.001	-0.023	0.83	0.000	-0.060	0.598	-0.651	0.71	0.000	30	13	1.56
	416	0.132	-0.138	0.89	0.000	0.001	-0.032	0.015	0.97	0.000	0.228	-0.438	0.467	0.96	0.000	28	NA	0.96
	418	0.050	0.098	0.64	0.000	0.153	0.007	-0.010	0.85	0.000	-0.018	0.316	-0.350	0.80	0.000	31	15	1.31
	420	0.282	-0.280	0.85	0.000	0.037	-0.081	0.027	0.90	0.000	0.482	-0.900	0.967	0.91	0.000	28	NA	0.89
	423	0.039	0.200	0.39	0.038	0.188	0.075	-0.016	0.73	0.000	-0.092	0.611	-0.648	0.80	0.000	29	12	1.42
	424	0.144	-0.041	0.93	0.000	0.144	-0.042	0.000	0.93	0.000	0.165	-0.108	0.103	0.93	0.000	28	NA	NA
	429	0.053	-0.033	0.91	0.000	0.047	-0.028	0.001	0.91	0.000	0.071	-0.088	0.086	0.93	0.000	29	NA	1.10
	430	0.096	0.125	0.74	0.000	0.275	-0.015	-0.020	0.93	0.000	-0.021	0.487	-0.558	0.88	0.000	27	14	1.54
	431	0.135	0.078	0.95	0.000	0.131	0.081	0.000	0.95	0.000	0.114	0.142	-0.100	0.95	0.000	27	NA	NA
	432	0.077	0.082	0.50	0.006	-0.097	0.227	0.019	0.68	0.000	0.098	0.016	0.103	0.50	0.024	29	5	1.18
	435	0.096	0.096	0.82	0.000	0.130	0.072	-0.004	0.83	0.000	0.035	0.281	-0.278	0.86	0.000	25	33	1.48
	436	0.141	0.030	0.99	0.000	0.145	0.026	0.000	0.99	0.000	0.136	0.045	-0.025	0.99	0.000	32	NA	NA
	437	0.035	0.050	0.78	0.000	0.070	0.017	-0.003	0.85	0.000	0.009	0.135	-0.139	0.85	0.000	33	23	1.18
	439	0.041	0.065	0.62	0.000	0.000	0.100	0.004	0.67	0.000	0.034	0.088	-0.037	0.62	0.002	30	0	1.11
	443	0.303	0.017	0.96	0.000	0.413	-0.072	-0.012	0.97	0.000	0.277	0.098	-0.126	0.96	0.000	28	34	2.65
	444	0.100	0.412	0.61	0.001	0.339	0.224	-0.027	0.86	0.000	-0.125	1.104	-1.067	0.95	0.000	27	13	2.10
	445	0.085	0.215	0.70	0.000	0.258	0.066	-0.018	0.92	0.000	-0.056	0.660	-0.708	0.94	0.000	30	14	1.64
	446	0.069	-0.009	0.96	0.000	0.092	-0.028	-0.002	0.97	0.000	0.064	0.007	-0.025	0.96	0.000	29	46	1.26
	**Mean**	0.108	0.073	0.76	0.024	0.140	0.047	-0.004	0.87	0.000	0.073	0.183	-0.171	0.87	0.001	29	35	1.50
$q = 2$	400	0.082	-0.052	0.87	0.000	0.033	-0.012	0.005	0.89	0.000	0.117	-0.162	0.173	0.89	0.000	29	NA	1.01
	401	0.213	-0.277	0.80	0.000	-0.125	0.014	0.035	0.96	0.000	0.423	-0.941	1.056	0.91	0.000	30	4	0.98
	402	0.035	-0.022	0.65	0.000	-0.038	0.042	0.007	0.88	0.000	0.066	-0.121	0.159	0.73	0.000	31	5	1.02
	403	0.042	-0.033	0.84	0.000	0.001	0.004	0.004	0.91	0.000	0.069	-0.121	0.142	0.90	0.000	32	NA	1.00
	404	0.164	0.068	0.88	0.000	0.184	0.050	-0.002	0.88	0.000	0.144	0.130	-0.100	0.88	0.000	30	92	2.01
	405	-0.017	0.148	0.19	0.317	0.102	0.043	-0.012	0.60	0.002	-0.121	0.480	-0.533	0.67	0.000	31	9	1.17
	406	0.132	-0.068	0.75	0.000	-0.029	0.074	0.016	0.85	0.000	0.223	-0.359	0.467	0.81	0.000	31	2	1.09
	407	0.001	0.460	0.00	0.982	0.126	0.358	-0.014	0.33	0.231	-0.206	1.100	-0.998	0.70	0.000	28	9	1.66
	408	0.125	0.008	0.92	0.000	0.064	0.059	0.007	0.94	0.000	0.149	-0.066	0.115	0.93	0.000	29	NA	1.12

	410	0.044	-0.044	0.85	0.000	0.002	-0.008	0.005	0.91	0.000	0.074	-0.138	0.148	0.90	0.000	29	NA	0.99
	411	0.088	0.495	0.45	0.014	0.406	0.228	-0.034	0.82	0.000	-0.172	1.307	-1.279	0.85	0.000	29	12	2.29
	412	-0.137	0.576	0.60	0.001	0.007	0.459	-0.016	0.65	0.001	-0.389	1.359	-1.220	0.84	0.000	28	0	1.56
	413	0.121	0.100	0.73	0.000	0.253	-0.007	-0.015	0.80	0.000	0.026	0.394	-0.459	0.79	0.000	28	17	1.58
	414	0.088	-0.018	0.95	0.000	0.111	-0.039	-0.002	0.95	0.000	0.088	-0.016	-0.004	0.95	0.000	32	56	1.34
	415	-0.009	0.228	0.10	0.586	0.151	0.091	-0.017	0.82	0.000	-0.132	0.619	-0.622	0.82	0.000	30	9	1.31
	416	0.146	-0.155	0.91	0.000	0.023	-0.055	0.014	0.96	0.000	0.251	-0.481	0.508	0.97	0.000	28	NA	0.93
	418	0.001	0.086	0.01	0.939	0.050	0.042	-0.005	0.53	0.010	-0.049	0.243	-0.253	0.69	0.000	31	10	1.11
	420	0.162	-0.086	0.90	0.000	0.116	-0.049	0.005	0.91	0.000	0.211	-0.239	0.237	0.92	0.000	28	NA	1.14
	423	0.016	0.120	0.23	0.232	0.110	0.042	-0.010	0.59	0.004	-0.061	0.362	-0.380	0.62	0.002	29	11	1.22
	424	0.108	-0.079	0.88	0.000	0.059	-0.040	0.005	0.90	0.000	0.160	-0.240	0.250	0.91	0.000	28	NA	1.03
	429	0.020	-0.014	0.82	0.000	0.010	-0.005	0.001	0.84	0.000	0.031	-0.047	0.051	0.86	0.000	29	NA	1.01
	430	0.078	0.121	0.66	0.000	0.250	-0.014	-0.020	0.90	0.000	-0.038	0.477	-0.549	0.84	0.000	27	13	1.44
	431	0.117	0.056	0.87	0.000	0.127	0.048	-0.001	0.87	0.000	0.106	0.091	-0.055	0.88	0.000	27	127	1.71
	432	0.010	0.137	0.10	0.613	-0.109	0.237	0.013	0.48	0.034	0.004	0.158	-0.034	0.10	0.868	29	8	1.12
	435	0.050	0.066	0.63	0.001	0.058	0.060	-0.001	0.63	0.004	0.011	0.184	-0.178	0.68	0.001	25	58	1.27
	436	0.202	-0.106	0.97	0.000	0.109	-0.022	0.009	0.99	0.000	0.273	-0.334	0.370	0.99	0.000	32	NA	1.08
	437	0.050	0.003	0.71	0.000	0.095	-0.039	-0.004	0.77	0.000	0.033	0.057	-0.088	0.73	0.000	33	24	1.18
	439	0.000	0.092	0.00	0.993	-0.014	0.104	0.001	0.15	0.743	-0.023	0.166	-0.117	0.32	0.240	30	14	1.08
	443	0.248	0.138	0.86	0.000	0.466	-0.040	-0.024	0.91	0.000	0.153	0.430	-0.456	0.88	0.000	28	19	2.40
	444	-0.008	0.573	0.04	0.834	0.309	0.325	-0.036	0.73	0.000	-0.318	1.526	-1.469	0.91	0.000	27	9	1.97
	445	0.083	0.271	0.52	0.003	0.347	0.043	-0.028	0.88	0.000	-0.117	0.901	-1.001	0.87	0.000	30	12	1.77
	446	0.074	-0.024	0.95	0.000	0.084	-0.033	-0.001	0.95	0.000	0.079	-0.040	0.025	0.95	0.000	29	84	1.29
	**Mean**	0.073	0.086	0.61	0.172	0.104	0.061	-0.004	0.79	0.032	0.033	0.209	-0.190	0.80	0.035	29	26	1.34
$q = 3$	400	0.087	-0.061	0.88	0.000	0.022	-0.007	0.007	0.92	0.000	0.131	-0.200	0.219	0.91	0.000	29	NA	0.99
	401	0.187	-0.234	0.79	0.000	-0.121	0.031	0.032	0.97	0.000	0.370	-0.813	0.921	0.90	0.000	30	4	0.99
	402	0.032	-0.023	0.67	0.000	-0.033	0.034	0.007	0.89	0.000	0.061	-0.116	0.150	0.75	0.000	31	5	1.02
	403	0.043	-0.031	0.84	0.000	0.001	0.008	0.004	0.91	0.000	0.071	-0.120	0.145	0.89	0.000	32	NA	1.01
	404	0.163	0.088	0.89	0.000	0.178	0.075	-0.002	0.89	0.000	0.134	0.180	-0.146	0.89	0.000	30	89	2.01
	405	-0.009	0.121	0.12	0.527	0.085	0.038	-0.010	0.54	0.008	-0.094	0.391	-0.433	0.63	0.001	31	9	1.14
	406	0.146	-0.100	0.77	0.000	-0.024	0.051	0.017	0.86	0.000	0.252	-0.437	0.542	0.83	0.000	31	1	1.07
	407	0.018	0.435	0.11	0.577	0.084	0.381	-0.007	0.20	0.590	-0.157	0.977	-0.844	0.58	0.005	28	12	1.66
	408	0.112	0.051	0.88	0.000	0.048	0.104	0.007	0.90	0.000	0.120	0.027	0.038	0.88	0.000	29	NA	1.15
	410	0.045	-0.039	0.87	0.000	0.009	-0.010	0.004	0.92	0.000	0.071	-0.121	0.128	0.91	0.000	29	NA	0.99
	411	0.076	0.525	0.38	0.042	0.422	0.236	-0.037	0.82	0.000	-0.204	1.402	-1.381	0.84	0.000	29	11	2.32
	412	-0.126	0.553	0.57	0.002	-0.018	0.466	-0.012	0.60	0.004	-0.355	1.263	-1.107	0.79	0.000	28	NA	1.61
	413	0.111	0.120	0.69	0.000	0.250	0.007	-0.016	0.77	0.000	0.008	0.441	-0.500	0.77	0.000	28	16	1.56
	414	0.103	-0.035	0.93	0.000	0.125	-0.054	-0.002	0.94	0.000	0.110	-0.057	0.037	0.93	0.000	32	63	1.40
	415	-0.025	0.272	0.28	0.131	0.131	0.137	-0.016	0.80	0.000	-0.162	0.704	-0.688	0.89	0.000	30	8	1.33
	416	0.171	-0.181	0.91	0.000	0.034	-0.069	0.015	0.96	0.000	0.293	-0.558	0.588	0.97	0.000	28	NA	0.92

	418	0.006	0.077	0.17	0.358	0.051	0.038	-0.005	0.53	0.010	-0.038	0.219	-0.229	0.68	0.000	31	10	1.11
	420	0.156	-0.039	0.91	0.000	0.155	-0.038	0.000	0.91	0.000	0.169	-0.079	0.062	0.91	0.000	28	NA	NA
	423	0.013	0.119	0.19	0.326	0.103	0.044	-0.010	0.57	0.005	-0.063	0.357	-0.374	0.62	0.002	29	10	1.20
	424	0.098	-0.067	0.89	0.000	0.056	-0.033	0.005	0.90	0.000	0.143	-0.207	0.217	0.91	0.000	28	NA	1.03
	429	0.022	-0.013	0.83	0.000	0.010	-0.003	0.001	0.85	0.000	0.032	-0.046	0.052	0.85	0.000	29	NA	1.01
	430	0.068	0.130	0.59	0.001	0.235	-0.001	-0.019	0.85	0.000	-0.053	0.501	-0.571	0.81	0.000	27	12	1.43
	431	0.119	0.047	0.85	0.000	0.124	0.042	-0.001	0.85	0.000	0.114	0.060	-0.021	0.85	0.000	27	124	1.67
	432	0.008	0.131	0.08	0.687	-0.114	0.233	0.013	0.51	0.021	0.008	0.130	0.002	0.08	0.923	29	9	1.10
	435	0.041	0.089	0.54	0.006	0.076	0.063	-0.004	0.57	0.013	-0.018	0.265	-0.265	0.67	0.002	25	19	1.23
	436	0.236	-0.141	0.95	0.000	0.088	-0.006	0.015	0.99	0.000	0.335	-0.460	0.518	0.98	0.000	32	NA	1.02
	437	0.088	-0.007	0.76	0.000	0.161	-0.075	-0.007	0.80	0.000	0.067	0.063	-0.115	0.76	0.000	33	23	1.31
	439	-0.010	0.107	0.25	0.184	-0.014	0.110	0.000	0.25	0.412	-0.041	0.204	-0.155	0.49	0.026	30	NA	0.00
	443	0.242	0.178	0.83	0.000	0.478	-0.013	-0.026	0.89	0.000	0.132	0.519	-0.531	0.85	0.000	28	18	2.46
	444	-0.042	0.605	0.22	0.279	0.265	0.365	-0.035	0.69	0.000	-0.362	1.589	-1.515	0.89	0.000	27	8	1.89
	445	0.090	0.292	0.50	0.005	0.390	0.033	-0.032	0.87	0.000	-0.131	0.988	-1.106	0.84	0.000	30	12	1.86
	446	0.097	-0.043	0.94	0.000	0.096	-0.042	0.000	0.94	0.000	0.114	-0.095	0.081	0.95	0.000	29	NA	NA
	**Mean**	0.074	0.091	0.63	0.098	0.105	0.067	-0.004	0.78	0.033	0.033	0.218	-0.196	0.80	0.030	29	26	1.36
$q = 4$	400	0.103	-0.078	0.87	0.000	0.015	-0.004	0.010	0.92	0.000	0.162	-0.262	0.290	0.91	0.000	29	NA	0.99
	401	0.180	-0.215	0.80	0.000	-0.112	0.037	0.031	0.97	0.000	0.351	-0.756	0.861	0.90	0.000	30	4	1.01
	402	0.034	-0.024	0.69	0.000	-0.032	0.034	0.007	0.90	0.000	0.065	-0.122	0.158	0.78	0.000	31	5	1.02
	403	0.048	-0.031	0.83	0.000	0.001	0.011	0.005	0.90	0.000	0.078	-0.129	0.158	0.88	0.000	32	NA	1.01
	404	0.164	0.108	0.89	0.000	0.180	0.094	-0.002	0.89	0.000	0.124	0.236	-0.203	0.90	0.000	30	90	2.06
	405	-0.002	0.106	0.02	0.896	0.079	0.035	-0.008	0.49	0.021	-0.076	0.343	-0.381	0.59	0.002	31	10	1.15
	406	0.167	-0.125	0.79	0.000	-0.015	0.037	0.019	0.87	0.000	0.286	-0.504	0.610	0.85	0.000	31	1	1.06
	407	0.049	0.403	0.28	0.151	0.063	0.392	-0.002	0.28	0.359	-0.095	0.851	-0.698	0.52	0.020	28	32	1.73
	408	0.105	0.091	0.85	0.000	0.055	0.133	0.005	0.87	0.000	0.094	0.126	-0.055	0.85	0.000	29	NA	1.22
	410	0.049	-0.040	0.88	0.000	0.014	-0.011	0.004	0.92	0.000	0.075	-0.121	0.128	0.91	0.000	29	NA	0.99
	411	0.076	0.533	0.37	0.049	0.432	0.234	-0.039	0.82	0.000	-0.211	1.430	-1.412	0.84	0.000	29	11	2.32
	412	-0.113	0.536	0.52	0.004	-0.029	0.468	-0.009	0.55	0.012	-0.326	1.200	-1.034	0.74	0.000	28	NA	1.58
	413	0.110	0.133	0.68	0.000	0.252	0.018	-0.016	0.77	0.000	0.002	0.469	-0.523	0.77	0.000	28	16	1.59
	414	0.117	-0.046	0.93	0.000	0.136	-0.064	-0.002	0.93	0.000	0.130	-0.088	0.068	0.93	0.000	32	68	1.45
	415	-0.033	0.308	0.33	0.075	0.137	0.162	-0.018	0.80	0.000	-0.187	0.795	-0.774	0.91	0.000	30	8	1.35
	416	0.187	-0.196	0.91	0.000	0.040	-0.077	0.016	0.95	0.000	0.318	-0.604	0.635	0.97	0.000	28	NA	0.92
	418	0.013	0.076	0.32	0.078	0.060	0.034	-0.005	0.59	0.002	-0.032	0.219	-0.231	0.69	0.000	31	12	1.13
	420	0.163	-0.019	0.90	0.000	0.179	-0.032	-0.002	0.90	0.000	0.161	-0.011	-0.012	0.90	0.000	28	90	1.81
	423	0.012	0.127	0.17	0.368	0.104	0.050	-0.010	0.57	0.006	-0.068	0.378	-0.395	0.63	0.001	29	10	1.21
	424	0.097	-0.058	0.89	0.000	0.059	-0.027	0.004	0.91	0.000	0.137	-0.183	0.195	0.92	0.000	28	NA	1.06
	429	0.026	-0.013	0.83	0.000	0.013	-0.002	0.001	0.85	0.000	0.037	-0.048	0.056	0.86	0.000	29	NA	1.02
	430	0.068	0.137	0.58	0.002	0.236	0.005	-0.019	0.83	0.000	-0.057	0.521	-0.592	0.80	0.000	27	12	1.44
	431	0.126	0.047	0.84	0.000	0.120	0.051	0.001	0.84	0.000	0.127	0.046	0.001	0.84	0.000	27	NA	1.54

432	0.015	0.120	0.14	0.464	-0.115	0.228	0.014	0.55	0.009	0.024	0.091	0.045	0.15	0.745	29	8	1.11
435	0.039	0.113	0.49	0.013	0.096	0.071	-0.007	0.58	0.012	-0.038	0.343	-0.347	0.70	0.001	25	14	1.25
436	0.260	-0.162	0.94	0.000	0.069	0.011	0.019	0.99	0.000	0.378	-0.542	0.617	0.97	0.000	32	NA	1.01
437	0.129	-0.023	0.79	0.000	0.223	-0.111	-0.009	0.83	0.000	0.106	0.051	-0.121	0.80	0.000	33	25	1.46
439	-0.012	0.118	0.29	0.120	-0.013	0.118	0.000	0.29	0.304	-0.047	0.227	-0.174	0.53	0.012	30	NA	0.00
443	0.249	0.198	0.82	0.000	0.492	0.001	-0.027	0.89	0.000	0.132	0.563	-0.568	0.85	0.000	28	18	2.55
444	-0.049	0.609	0.25	0.210	0.240	0.383	-0.033	0.66	0.001	-0.365	1.579	-1.494	0.88	0.000	27	7	1.86
445	0.095	0.303	0.50	0.005	0.416	0.027	-0.034	0.87	0.000	-0.135	1.030	-1.157	0.83	0.000	30	12	1.92
446	0.120	-0.060	0.94	0.000	0.111	-0.052	0.001	0.94	0.000	0.145	-0.140	0.127	0.95	0.000	29	NA	1.35
**Mean**	0.081	0.093	0.64	0.076	0.110	0.070	-0.003	0.78	0.023	0.040	0.218	-0.194	0.80	0.024	29	37	1.43
